# Isoprenylcysteine Carboxyl Methyltransferase and Its Substrate Ras Are Critical Players Regulating TLR-Mediated Inflammatory Responses

**DOI:** 10.3390/cells9051216

**Published:** 2020-05-14

**Authors:** Woo Seok Yang, Han Gyung Kim, Eunji Kim, Sang Yun Han, Nur Aziz, Young-Su Yi, Sunggyu Kim, Yunmi Lee, Byong Chul Yoo, Jeung-Whan Han, Narayanan Parameswaran, Ji Hye Kim, Jae Youl Cho

**Affiliations:** 1 Department of Integrative Biotechnology, Biomedical Institute for Convergence at SKKU, Sungkyunkwan University, Suwon 16419, Korea; real0902@gmail.com (W.S.Y.); hanks523@skku.edu (H.G.K.); im144069@gmail.com (E.K.); dangsukr@naver.com (S.Y.H.); nuraziz.20@gmail.com (N.A.); 2 Department of Life Science, Kyonggi University, Suwon 16227, Korea; ysyi@kgu.ac.kr; 3 Research and Business Foundation, Sungkyunkwan University, Suwon 16419, Korea; sukim590@skku.edu; 4 Department of Chemistry, Kwangwoon University, Seoul 01897, Korea; ymlee@kw.ac.kr; 5 Division of Translational Science, Research Institute, National Cancer Center, Goyang 10408, Korea; yoo_akh@ncc.re.kr; 6 Research Center for Epigenome Regulation, School of Pharmacy, Sungkyunkwan University, Suwon 16419, Korea; jhhan551@skku.edu; 7 Department of Physiology and Division of Pathology, Michigan State University, East Lansing, MI 48824, USA; narap@msu.edu

**Keywords:** Ras, isoprenylcysteine carboxyl methyltransferase, inflammation, TIR domain, AP-1 pathway

## Abstract

In this study, we investigated the functional role of isoprenylcysteine carboxyl methyltransferase (ICMT) and its methylatable substrate Ras in Toll-like receptor (TLR)-activated macrophages and in mouse inflammatory disease conditions. *ICMT* and *RAS* expressions were strongly increased in macrophages under the activation conditions of TLRs by lipopolysaccharide (LPS, a TLR4 ligand), pam3CSK (TLR2), or poly(I:C) (TLR3) and in the colons, stomachs, and livers of mice with colitis, gastritis, and hepatitis. The inhibition and activation of ICMT and Ras through genetic and pharmacological approaches significantly affected the activation of interleukin-1 receptor-associated kinase (IRAK)s, tumor necrosis factor receptor associated factor 6 (TRAF6), transforming growth factor-β-activated kinase 1 (TAK1), mitogen-activated protein kinase (MAPK), and MAPK kinases (MAPKKs); translocation of the AP-1 family; and the expressions of inflammation-related genes that depend on both MyD88 and TRIF. Interestingly, the Ras/ICMT-mediated inflammatory reaction critically depends on the TIR domains of myeloid differentiation primary response 88 (MyD88) and TIR-domain-containing adapter-inducing interferon-β (TRIF). Taken together, these results suggest that ICMT and its methylated Ras play important roles in the regulation of inflammatory responses through cooperation with the TIR domain of adaptor molecules.

## 1. Introduction

Macrophages are innate immune cells involved in homeostatic and pathophysiological processes, including the development of inflammatory diseases. Macrophages express pattern-recognition receptors (PRRs) that recognize pathogen-associated molecular patterns (PAMPs) presented by microbes or damage-associated molecular patterns (DAMPs) released from damaged cells. Activation of macrophage PRRs by PAMPs and DAMPs is now known to be an important factor in the pathogenesis of inflammatory diseases. A representative family of PRRs is Toll-like receptors (TLRs), which are type I transmembrane proteins [1,2]. The activation of TLRs by various ligands and DAMPs initiates inflammatory signaling cascades via adaptor molecules such as myeloid differentiation factor 88 (MyD88) and Toll-interleukin (IL)-1 receptor domain–containing adaptor molecule-1 (TRIF) [3,4]. MyD88 and TRIF have structurally identical domains, including the Toll/IL-1R (TIR) domain. Different TLRs use different adaptor proteins; TLR4 uses both MyD88 and TRIF, TLR3 uses only TRIF, and the other TLRs generally use MyD88. Upon TLR stimulation, a carboxyl (C)-terminal TIR domain forms a homodimer via a TIR–TIR domain interaction, which leads to the formation of an intracellular signaling complex that activates downstream signaling, including the activation of major transcription factors such as activator protein (AP)-1, nuclear factor (NF)-κB, and cAMP response element-binding protein (CREB) [5].

AP-1 is a key transcription factor regulated by mitogen activated protein kinase (MAPK) signaling pathways that plays key roles in inflammation and cancer [6]. Stimulation of MyD88 and TRIF by a specific PAMP (i.e., lipopolysaccharide [LPS]) can degrade interleukin-1 receptor-associated kinase (IRAK) 1/4 and tumor necrosis factor (TNF) receptor-associated factor 6 (TRAF6), leading to the induction of AP-1 activation signaling cascades via transforming growth factor β-activated kinase 1 (TAK1); MAPK kinases (MAPKKs), including MEK1/2, MKK3/6, and MKK4/7; and MAPKs (extracellular signal-regulated kinase 1/2 [ERK1/2], c-Jun N-terminal kinase [JNK], and p38). In addition, TLR-induced AP-1 activation has been shown to involve small GTPases such as rat sarcoma (Ras), the Ras homolog (Rho) family, and raf proto-oncogene serine/threonine-protein kinase (c-Raf) [7,8]. Even though links between Ras, AP-1, and inflammatory signaling have been detected in previous studies, the specific mechanisms by which TLR-induced Ras activation occurs are not well-known.

Members of the Ras protein family play essential roles in a variety of biological processes associated with cell cycle regulation and are therefore crucial signaling proteins in cell survival, proliferation, gene expression, and carcinogenesis [9]. The role of Ras has also been explored in various inflammatory conditions. *Helicobacter pylori*-induced expressions of COX-2 and iNOS are mediated by Ras in gastric epithelial AGS cells [10]. Furthermore, the expression of mutant K-Ras in K19(+) gastric epithelial cells induces chronic inflammation and promotes the development of dysplasia [11]. Other studies have shown that LPS-induced TNF-α production involves Ras/Raf-1/MEK/MAPK [12]. In line with those studies, Ras has been shown to be highly expressed in dextran sodium sulfate (DSS)–induced colitis, and its suppression by farnesylthiosalicylic acid reduces the severity of colitis, suggesting that Ras is a therapeutic target in inflammatory disease [13]. Because of its role in cell cycle regulation, several clinical trials have used farnesyltransferase inhibitors to block Ras modification and thereby block cancer progression. However, even though farnesylation was blocked, Ras activity was maintained via geranylgeranylation, limiting the benefits of targeting Ras modification with a lipid moiety [14,15,16,17,18]. Thus, a better understanding of Ras modifications and how they affect Ras activity could improve Ras-targeted therapeutic approaches.

Ras activity to interact with GTP exchanging factors (GEFs) is critically determined by post-translational modification of the C-terminal CAAX motif [19,20]. Biochemical modification of Ras allows its plasma membrane translocation, which involves three steps. The first step is protein farnesylation or geranylgeranylation, during which farnesyl or geranylgeranyl isoprenoid lipids covalently bond to the cysteine residue of the CAAX proteins via farnesyl transferase or geranylgeranyl transferase, respectively. Ras proteins are farnesylated, and GTP-binding proteins are geranylgeranylated by the appropriate transferase [21,22]. During the second step, the three C-terminal amino acids (-AAX) are cleaved by CAAX prenyl protease 2 (RCE1) [23]. Third, the prenylated cysteine is methylated by protein-*S*-isoprenylcysteine *O*- methyltransferase (ICMT) [24], which is a polytopic membrane protein that spans the ER membrane eight times [25,26]. The methylation of Ras is essential for its palmitoylation rendering it in plasma membrane localization of Ras [27,28], which causes propagation of Ras-mediated signaling processes for various cellular responses [29,30].

ICMT is of therapeutic interest for selectively inhibiting Ras activity. Because ICMT catalyzes Ras modification as a polytopic membrane protein that spans the ER membrane eight times [25] for a final step in membrane anchoring [31], inhibiting ICMT could suppress the translocation of Ras to the membrane and thus inhibit Ras activity [32]. Indeed, ICMT inhibitors have been proposed for treating Ras-associated tumorigenic responses in various cancers [33]. Unlike farnesyltransferase inhibition, ICMT inhibition could selectively suppress Ras without compensation by other enzymes [22,34,35,36]. Although other studies have examined the role of Ras in inflammation [37] and the role of ICMT in Ras activity, the relationship between ICMT-mediated Ras activation and inflammation remains largely unexplored. Moreover, only few papers stating that ICMT regulates inflammatory responses have been reported [38]. In this report, therefore, we focus on understanding the functional involvement of ICMT and Ras in inflammatory responses, including intracellular signaling, inflammatory gene expression, and pathophysiological symptoms mediated by TLRs and their adaptor molecules, such as MyD88 and TRIF. We demonstrate that the ICMT pathway is a potentially important therapeutic target for inflammatory diseases. 

## 2. Materials and Methods

### 2.1. Mice and Reagents

Six-week-old ICR, Balb/c, and C57BL/6 male mice (see Table S2 for genetic background information) were purchased from Daehan Biolink (DBL, Chungbuk, Korea) and housed eight mice per group under a 12-h light/dark cycle (lights on at 6 a.m.). Water and a pellet diet (Samyang, Daejeon, Korea) were supplied ad libitum. Animal care followed guidelines issued by the National Institutes of Health for the Care and Use of Laboratory Animals (NIH Publication 80-23, revised in 1996) and the Institutional Animal Care and Use Committee at Sungkyunkwan University (Approval No.: SKKUIACUC-2016-01-0002-2). Phorbol-12-myristate, sodium carboxyl methylcellulose, 2,4-dinitrochlorobenzene (DNCB), dextran sodium sulphate (DSS), acetylsalicylic acid, D-GalN, LPS (*E. coli* 0111:B4), 100% EtOH, and HCl were purchased from Sigma Chemical Co. (St. Louis, MO, USA). MAPK inhibitors (SB203580, SP600125, and U0126) were purchased from Calbiochem (La Jolla, CA, USA). RAW264.7, HEK293, and MDA-MB-231 cells were purchased from the American Type Culture Collection (Manassas, VA, USA). Detailed information on antibodies used in this study is explained in Supplementary Materials. The AP-1 luciferase construct was purchased from Addgene (Cambridge, MA, USA).

### 2.2. Construction of Expression Vectors

GFP-tagged wild type ICMT (Uniprot ID: O60725) construct (*Flag-GFP-ICMT-WT*) was obtained from the National Cancer Center (Goyang, Korea). Mutant E167 (*Flag-EGFPc1-ICMT E167*) was created using the QuickChange site-directed mutagenesis kit (Stratagene) with the following primers, *E167A* forward [F]-5’- CGC GAT CGA ACA GAA GCA GAA ATC TCA CTA ATT CAC-3’ and reverse [R]-5’- GTG AAT TAG TGA GAT TTC TGC TTC TGT TCG ATC GCG -3’, using the manufacturer’s *Flag-EGFPc1-ICMT-WT* as a template. The *ICMT* luciferase construct was constructed using *Flag-EGFPc1-ICMT-WT*. *MyD88* and *TRIF* plasmids were purchased from Addgene (Cambridge, MA, USA). We constructed *MyD88* mutant plasmids (Δ*TIR*, Δ*Y58F*, Δ*ITAM*, Δ*DD,* and Δ*ID*) and *TRIF* mutant plasmids (Δ*TIR*) from *MyD88* and *TRIF* plasmids using site-directed mutagenesis. Briefly, target primers for each mutant plasmid were designed, and PCR was performed with *pfu* polymerase. PCR parameters were as follows: pre-denaturation (95 °C, 30 s) and then 18 cycles of denaturation (95 °C, 30 s), annealing (55 °C, 1 min), and elongation (68 °C, 1 min/kb). We transformed the PCR products into DH5α competent cells (Invitrogen, Carlsbad, CA, USA) and cultured the transformed cells on LB agar plates containing ampicillin (100 mg/mL) at 37 °C for 16 h. We confirmed all constructs by automated DNA sequencing.

### 2.3. Preparation of Peritoneal Macrophages

Peritoneal exudates were extracted from ICR mice (6-weeks-old, 17 to 21 g) by lavage 4 days after intraperitoneal treatment with 4% thioglycollate broth (Difco Laboratories, Detroit, MI, USA). After the blood was removed from the exudates using RBC lysis buffer (Sigma Chemical Co.), the extracted peritoneal macrophages (1 × 10^6^ cells/mL) were plated in a 100 mm tissue culture plate and incubated for 4 h at 37 °C in a 5% CO_2_ humidified atmosphere.

### 2.4. Cell Culture and Drug Preparation

Murine macrophage-like RAW264.7 cells, MDA-MB-231 cells, and primary cells (peritoneal macrophages) were cultured in RPMI 1640 medium (Gibco, Grand Island, NY, USA) containing 100 U/mL penicillin, 100 µg/mL streptomycin, and 10% FBS (Gibco). Human embryonic kidney 293 (HEK293) cells were maintained in DMEM medium (Gibco) with antibiotics (penicillin and streptomycin) and FBS. Cells were grown at 37 °C and 5% CO_2_ in a humidified atmosphere. Cysmethynil (CyM), an ICMT inhibitor, was purchased from EMD Millipore (Billerica, MA, USA). ICMT inhibitors used in in vivo experiments were prepared using ethanol, polyethylene glycol 400, and 5% dextrose at a 1:6:3 ratio. For the in vitro study, ICMT inhibitors were dissolved in 100% dimethyl sulfoxide (DMSO).

### 2.5. Induction and Monitoring of DNCB-Induced Atopic Dermatitis (AD) in Mice

An AD mouse model was created by administering DNCB to NC/Nga mice (Daehan Biolink, Osong, Korea), as previously described [39]. Briefly, 1% DNCB (200 µL) in acetone/olive oil (3:1) was applied for sensitization to DNCB. After three days, 0.4% DNCB (200 µL) was reapplied to shaved skin of the dorsal area two times per week for four weeks. Symptom severity in the AD mice was assessed every week.

### 2.6. Induction of Ulcerative Colitis

Acute colitis was induced in C57BL/6 mice (n = 7/group) through oral administration of 3% DSS (w/v) in fresh tap water ad libitum for 7 days. The phenotype of ulcerative colitis was measured by the length of colonic tissue on day 7 in the afternoon. The same protocol was carried out twice independently.

### 2.7. EtOH/HCl-Induced Gastritis

Acute gastritis was induced with EtOH/HCl according to a published method [40]. We orally administered 400 µL of 60% ethanol in 150 mM HCl to fasted ICR mice. Each animal was anaesthetized and sacrificed by urethane overdose 1 h after administration of necrotizing agent. The stomach was dissected and gently rinsed with phosphate-buffered saline (PBS).

### 2.8. LPS-Induced Hepatitis Mouse Model

Acute animal hepatitis was produced by LPS injection. Balb/C mice were given intraperitoneally with LPS (5 µg/kg) and D-galactosamine (800 mg/kg) to stimulate hepatitis. Each animal was anesthetized by urethane overdose 1 h after administration of the hepatitis stimulus, and blood was extracted from the portal vein. After liver extraction, liver tissue was rinsed in PBS. Serum was separated from blood by centrifugation at 600× *g* for 15 min. Levels of serum ALT and AST were measured with a Roche Modular spectrophotometric autoanalyzer.

### 2.9. LPS-Induced Septic Shock Mouse Model

Acute animal septic shock was produced by LPS injection. Balb/c mice were intraperitoneally injected with LPS (10 mg/kg). After 24 h, mice were sacrificed, and some immune organs (spleen, thymus, and lung) were analyzed to determine the level of ICMT.

### 2.10. Histopathology

Histopathological examinations were performed, as previously described [41,42]. Liver and colon tissues were stained with hematoxylin and eosin and examined for signs of tissue injury under a photomicroscope.

### 2.11. Generation of Stable Cell Lines

RAW 264.7 cells stably expressing GFP-tagged ICMT were maintained in RPMI 1640 containing 10% fetal bovine serum, and 100 µg/mL streptomycin, with Geneticin^®^ (G418, ThermoFisher Scientific, Waltham, MA, USA) and kanamycin were used as selectable markers.

### 2.12. CRISPR-Cas9-Mediated Depletion of ICMT

To delete the *ICMT* gene in RAW264.7 cells, we co-transfected cells with pc3-U6-guide *RNA*-*CMV*-*RED* (encoding gRNA and red fluorescent protein) and *Cas9*-*IRES*-*EGFP* (encoding Cas9 and green fluorescent protein) plasmids (gifts from Shanghai Biomodel Organism Science and Technology Development Co., Shanghai, China) using lipofectamine 3000 (Invitrogen, Carlsbad, CA). Four target sequences for gRNA synthesis were tested: 50-CACCGGGGCTGAGTGCGGAGGGACC-30, 50-CGGTCCCTCCGCACTCAGCCCCAAA-30. After ligation of the synthesized sequences into pc3-U6-guide *RNA-EGFP* and co-transfection of the two plasmids into RAW264.7 cells, cells with both red and green fluorescence were sorted using a Gallios flow cytometer (Beckman Coulter, La Brea, CA, USA). Sorted cells were cultured for 3 to 5 days, and clones propagated from a single cell were selected. Depletion of ICMT was confirmed by both western blotting and DNA sequencing.

### 2.13. RNA Interference

ICMT small interfering RNA (siRNA) duplexes were synthesized by Macrogen (Seoul, Korea), and 21-nucleotide sequences of siRNAs were synthesized for transient knockdown (Table 1). We transfected ICMT siRNA into RAW264.7 cells using Lipofectamine^®^ RNAiMAX and a standard protocol (ThermoFisher Scientific). Forty-eight hours after transfection with siRNA, we determined the efficiency of RNA interference using Q-PCR and western blot assays.

### 2.14. mRNA Analysis by Semi-Quantitative or Quantitative Reverse Transcriptase-Polymerase Chain Reaction

Total RNA was isolated from animal tissues and cell lines including RAW264.7, MDA-MB-231, and HEK293 cells using TRIzol Reagent (Gibco) to identify inflammatory gene expression. RNA was prepared according to the manufacturer’s instructions. cDNA was synthesized from RNA using reverse transcriptase, and real-time PCR was performed on a real-time thermal cycler (Bio-Rad, Hercules, CA, USA), as reported previously [43]. Results were analyzed as the ratio of optimal density relative to glyceraldehyde 3-phosphate dehydrogenase (GAPDH). All primers used in the experiments were synthesized by Macrogen (Seoul, Korea) and are listed in Table 2.

### 2.15. Preparation of Cell Lysates and Nuclear Fractions from Cells/Tissues for Immunoblotting Analyses

Whole lysates were extracted from animal tissues, RAW264.7 cells, and HEK293 cells. Cells and tissues were washed with PBS, lysis buffer was added, and samples were lysed to prepare whole cell lysates as described in detail in the Supplementary Materials section.

### 2.16. Immunoprecipitation Assays

We performed immunoprecipitation assays to determine the binding activities of proteins as described in detail in Supplementary Materials.

### 2.17. Luciferase Reporter Gene Activity Assay

After plating 1 × 10^6^ HEK293 and RAW264.7 cells/mL in 12-well plates, we transfected the cells with *AP-1-Luc*, *ICMT-Luc*, *β-galactosidase*, *MyD88*, *MyD88* mutants, *TRIF*, or *TRIF* mutants using a Lipofectamine 3000 (ThermoFisher Scientific). After 24 h, we treated the cells with inhibitors as needed and lysed the sample with reporter lysis buffer. Lysates were centrifuged at 12,000 rpm for 10 min, and then 20 µL of the supernatant fraction and 20 µL of luciferase substrate were added. Following incubation, luciferase activity was measured using a luminescence spectrometer. Luciferase activity was normalized to β-galactosidase activity.

### 2.18. Confocal Microscopy

We performed confocal microscopy analysis to determine localization pattern of ICMT and Ras in ER as described in detail in the Supplementary Materials section.

### 2.19. Microarray Analysis

After preparing total RNA from RAW264.7 cells treated and untreated with LPS and from RAW264.7-*ICMT^−/−^* cells treated and untreated with LPS, we used the GeneChip^®^ Mouse Gene 2.0 ST Array as a platform. Detailed analysis method is explained in the Supplementary Materials section.

### 2.20. ICMT Activity Assay

A non-radioactive colorimetric continuous enzyme kit (#786-430, G-Biosciences, San Jose, CA, USA) was used to measure the enzyme activity of ICMT. We added 5 μL of SAM methyltransferase assay buffer in the blank group, 5 µL of sample transfected with *ICMT* or *ICMT E167A* in the sample group, and 5 µL of ICMT substrate (N-acetyl- S-farnesyl-L-cysteine, Sigma). We added 10 µL of ICMT inhibitor (CyM) as needed. Then, 100 µL of SAM Methyltransferase Master Mix (SAM Methyltransferase Assay Buffer, SAM Enzyme Mix, SAM Colorimetric Mix, S-adenosylhomocysteine) was added to all wells. We used a kinetic spectrophotometer at an absorbance wavelength of 510 nm and 37 °C once every 30 s for 30 min.

### 2.21. Statistical Analyses

All data presented in this paper are expressed as mean ± SD. Detailed analysis method is explained in the Supplementary Materials section.

## 3. Results

### 3.1. ICMT Is Highly Increased in TLR-Mediated Inflammatory Responses in Macrophages and in Mouse Disease Models

**Figure 1:**
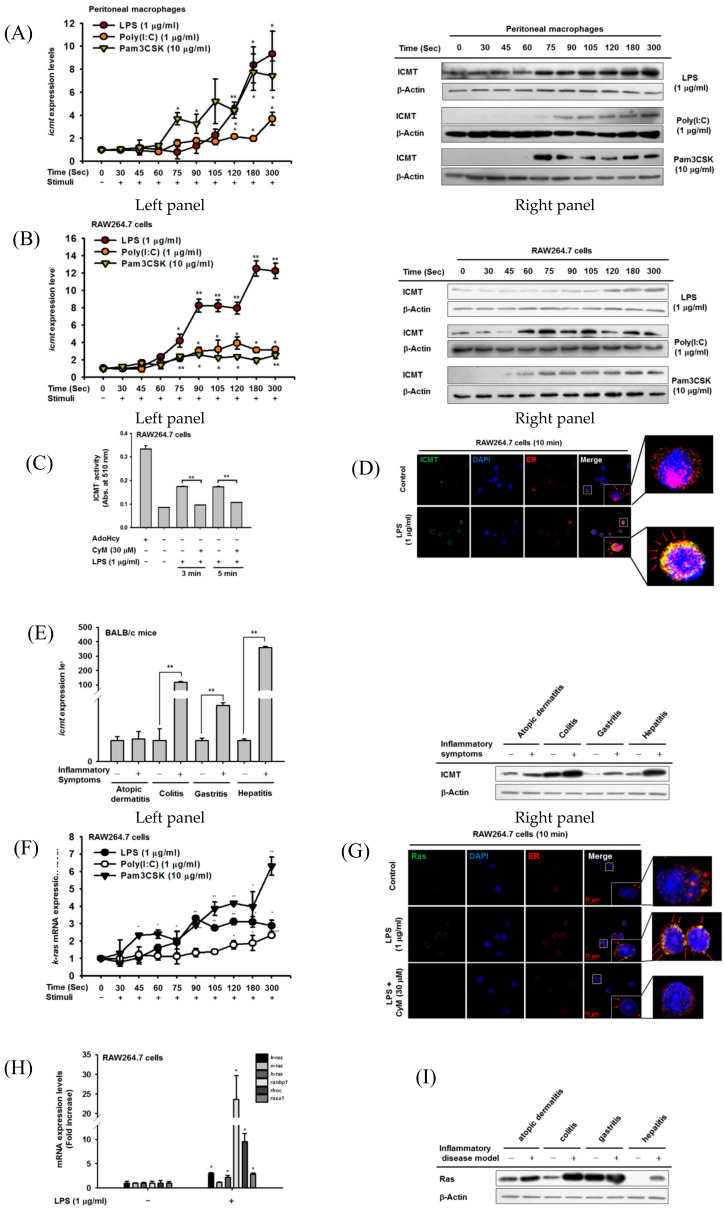
ICMT is highly expressed in chronic inflammatory conditions and TLR-mediated macrophage activation conditions. (**A**,**B**) The mRNA expression (left panel) of *ICMT* and protein level (right panel) of ICMT in peritoneal macrophages and RAW264.7 cells treated with LPS (1 µg/mL), poly(I:C) (1 µg/mL), and pam3CSK (10 µg/mL) were determined by real-time PCR and immunoblotting analysis, respectively. Protein (right panel) and mRNA expression (left panel) levels of ICMT in activated macrophages were determined by immunoblotting analysis and real-time PCR, respectively. (**C**) ICMT enzyme activity was measured (using a non-radioactive colorimetric continuous enzyme kit) in whole cell lysates of RAW264.7 cells treated with LPS (1 µg/mL) in the presence or absence of CyM (30 µM). (**D**) ER trafficking of ICMT was identified by confocal microscopy of RAW264.7 cells stimulated with LPS (1 µg/mL) for 10 min. (**E**) mRNA (left panel) and protein expressions (right panel) of *ICMT* in representative inflammatory disease animal models were determined by immunoblotting analysis and real-time PCR, respectively. Atopic dermatitis, colitis, gastritis, and hepatitis were induced by 2% DNFB, 3% DSS, HCl/EtOH, and LPS (5 mg/kg)/D-GalN (800 mg/kg), respectively. (**F**) The mRNA levels of *K-RAS* of RAW264.7 cells treated with 1 µg/mL of LPS, 1 µg/mL of poly(I:C), or 10 µg/mL of pam3CSK were determined by real-time PCR. (**G**) ER trafficking of Ras was identified by confocal microscopy in RAW264.7 cells stimulated with LPS (1 µg/mL) for 10 min in the presence or absence of CyM. (**H**) The Ras related genes (*K-RAS, N-RAS, H-RAS, RANBP1, RHOC*, and *RASA1*) of RAW264.7 cells treated with 1 µg/mL of LPS were determined by real-time PCR. (**I**) Protein levels of *K-RAS* in representative inflammatory disease animal models were determined by immunoblotting analysis. Atopic dermatitis, colitis, gastritis, and hepatitis were induced by 2% DNFB, 3% DSS, HCl/EtOH, and LPS (5 mg/kg)/D-GalN (800 mg/kg), respectively. *: *p* < 0.05 and **: *p* < 0.01 compared to the normal or LPS-treated groups. AdoHcy: 2-S-adenosyl-1-homocysteine.

To evaluate the effects of TLR stimulation on ICMT expression and activity, we treated peritoneal macrophages or macrophage-like RAW264.7 cells with LPS, pam3CSK, and Poly(I:C), known ligands of TLR4, TLR2, and TLR3, respectively [44]. The ligands significantly increased *ICMT* mRNA (Figure 1A, left panel) and ICMT protein levels (Figure 1A, right panels) in the peritoneal macrophages. Those effects were time-dependent, with a significant increase in mRNA levels seen 1 min after LPS stimulation and a clear increase in ICMT protein levels from 2 min. Similar time-dependent changes were seen upon treatment with Poly(I:C) and pam3CSK (Figure 1A). However, there was no alteration of ICMT level during normal culture conditions of RAW264.7 cells (Figure S1). Those effects were also observed in RAW264.7 cells; *ICMT* mRNA and ICMT protein levels were found to be increased in cancerous macrophages (Figure 1B). Consistent with this increase, LPS treatment enhanced the enzymatic activity of ICMT prepared by immunoprecipitation from lysates of LPS-treated RAW264.7 cells at 3 and 5 min (Figure 1C). Treatment with a pharmacological ICMT inhibitor (CyM, 30 µM) (Figure S2) strongly reduced the enzyme activity of the immunoprecipitated ICMT (Figure 1C). More intriguingly, LPS treatment for 10 min increased the endoplasmic reticulum (ER) trafficking of ICMT (Figure 1D). Since mRNA and protein levels of ICMT were strongly induced in TLR-activated macrophages, we next examined whether ICMT is also increased in in vivo inflammatory diseases. To prepare mouse models of inflammation, we used several well-known pathological conditions including atopic dermatitis triggered by 2,4-dinitrofluorobenzene (DNFB), colitis induced by DSS, gastritis generated by HCl/EtOH, and hepatitis induced by LPS/D-galactosamine (D-GalN), as reported previously [40,43,45,46] and measured levels of *icmt* mRNA and ICMT protein in these disease models. As shown in Figure 1E, the mRNA level of *icmt* was significantly increased compared to normal conditions (left panel). Consistent with the mRNA level, the ICMT protein level was also upregulated in the colitis, gastritis, and hepatitis conditions compared to normal conditions (Figure 1E, right panel). Moreover, we also evaluated human disease data from NCBI Gene Expression Omnibus. *icmt* mRNA in various human chronic inflammatory diseases was revealed to be elevated in patient groups with inflammatory-dilated cardiomyopathy (IC), rheumatoid arthritis (RA), Crohn disease (CD), ulcerative colitis (UC), and inflammatory bowel disease (IBD) in ileum (I) and colon (C) compared to healthy groups, with an up to three-fold-increase observed in inflammatory bowel diseases (Figure S3). Meanwhile, since ICMT is known to be an enzyme modifying plasma membrane translocation of Ras [47], we next examined the expression pattern of Ras in LPS-treated RAW264.7 macrophages. As shown in Figure 1F, *K-RAS* level was also time-dependently increased under exposure to 1 µg/mL of LPS, 200 µg/mL of poly(I:C), and 1 µg/mL of Pam3CSK. Similar to ICMT, LPS stimulation increased Ras expression in the ER, which was strongly inhibited by treatment with 30 µM of CyM (Figure 1G). In addition, mRNA levels of the Ras family such as *K-RAS*, *N-RAS*, *H-RAS*, *RANBP1*, and *RAS-A1* were also examined, simultaneously. As Figure 1H depicts, there was no induction in N-RAS, whereas others were increased from 2- to 20-fold by LPS stimulation. Moreover, similar to ICMT level in various mouse inflammatory disease models, Ras was also found to be increased in each disease state (Figure 1I). In particular, Ras displayed clear induction level in intestines and livers of colitis- and hepatitis-bearing mice.

### 3.2. ICMT Regulates Inflammatory Response In Vitro

**Figure 2:**
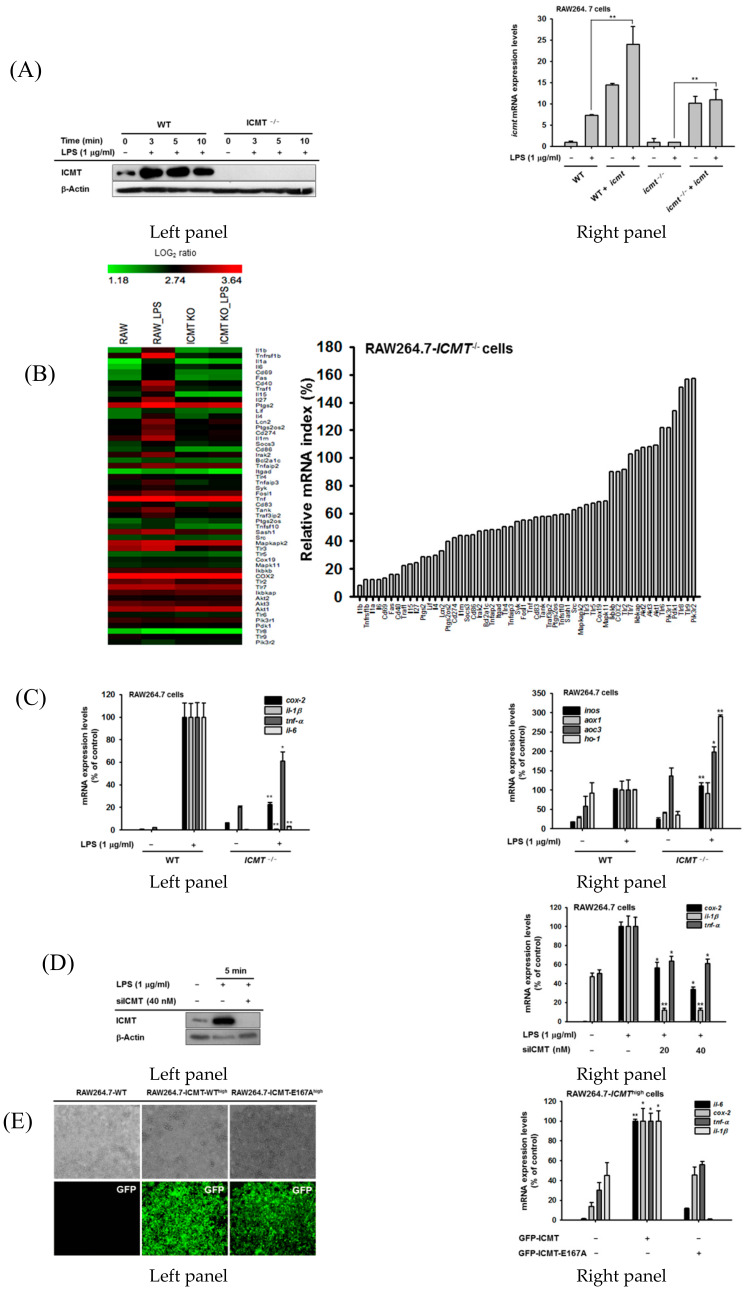
Functional and expressional suppression of ICMT reduces inflammatory responses in vitro and in vivo. ((**A**), left panel) Protein levels of *ICMT* in RAW264.7-*ICMT*^−/−^ cells (constructed using the CRISPR/Cas9 system) during LPS (1 µg/mL) treatment. ((**A**), right panel) mRNA levels of *ICMT* in RAW264.7-*ICMT*^−/−^ cells during LPS (1 µg/mL) exposure after transfection with *ICMT* plasmid. (**B**) A microarray analysis was used to compare the expressions of 54 inflammatory genes between RAW264.7-wild type cells and RAW264.7-*ICMT*^-/-^ cells treated with LPS (1 µg/mL) was carried out. (**C**) mRNA expression levels of pro-inflammatory genes (left panel), such as *COX-2, IL-1β, IL-6,* and *TNF-α*, and radical-generating genes (right panel), including *INOS, AOX1, AOC3,* and *HO-1*, were measured using real-time PCR in RAW264.7-wild type cells and RAW264.7-*ICMT*^−/−^ cells treated with LPS (1 µg/mL). ((**D**), left panel) Knockdown levels of *ICMT* in LPS-treated RAW264.7 cells treated with siRNA to *ICMT* (siICMT). ((**D**), right panel) mRNA expression levels of pro-inflammatory genes, such as *COX-2, IL-1β,* and *TNF-α* were measured in RAW264.7-wild type cells treated with LPS (1 µg/mL) in the presence or absence of siICMT using real-time PCR. (**E**, left panel) Fluorescence microscopic analysis of RAW264.7 cells expressing high levels of *GFP-ICMT-wild type* (*WT*) or its dominant mutation form, *GFP-ICMT-E167A*. (**E**, right panel) The mRNA expression levels of pro-inflammatory genes including *IL-6, COX-2, IL-1β,* and *TNF-α* were measured using real-time PCR in RAW264.7-*ICMT*^high^ cells and -*ICMT-E167A*^high^ cells. *: *p* < 0.05 and **: *p* < 0.01 compared to the LPS-treated group or vector control.

Based on the data just described, we next examined whether inhibition of *ICMT* expression and function is correlated with a reduction in inflammatory responses in vitro. For this purpose, we established *ICMT* knockout RAW264.7 (RAW264.7-*ICMT^−/−^* cells) macrophages using CRISPR-Cas9 without displaying any induction of ICMT by LPS (Figure 2A, left panel), as reported previously [48]. To rule out off-target effects of the knockout (KO) strategy, we also overexpressed *ICMT* in *ICMT* KO cells (Figure 2A, right panel). The *ICMT* KO and control cell lines were stimulated with lipopolysaccharide (LPS), and mRNA expression of the inflammatory genes was assessed by microarray analysis. LPS-treated *ICMT^−/−^* cells were defective in the mRNA expression of pro-inflammatory cytokines including *IL-1β*, *IL-1α*, *TNF-α*, and *IL-6* (Figure 2B). Levels of co-stimulatory molecules such as *CD86*; pattern recognition molecules including *TLR4*, *TLR3*, and *TLR5*; and signaling molecules involved in inflammatory responses including *SYK*, *SRC*, and *TANK* also decreased in the *ICMT* knockout condition (Figure 2B). Expressions of some of these genes were confirmed using real-time polymerase chain reaction (PCR): levels of *COX-2*, *IL-1β*, and *TNF-α* were confirmed to be reduced in *ICMT*^−/−^ cells compared to controls (Figure 2C, left panel). Interestingly, genes for enzymes that generate radicals including *INOS*, *AOX1*, *AOC3*, and *HO-1* were significantly enhanced in the *ICMT*^−/−^ cells (Figure 2C, right panel). Consistent with the CRISPR knockout cells, siRNA-mediated knockdown of *ICMT* (Figure 2D, left panel) also inhibited LPS-induced expression of *COX-2*, *IL-1β*, and *TNF-α* compared to control cells (Figure 2D, right panel). Consistent with the effects of ICMT suppression on inflammatory genes, overexpression of *ICMT* in RAW264.7 (*ICMT-WT^high^*) macrophages (Figure 2E, left panel) resulted in enhanced expressions of *COX-2*, *IL-1β*, and *TNF-α*(Figure 2E, right panel). In contrast, as expected, the transcripts were attenuated in high expression cell line (*ICMT-E167A^high^*-expressing cells) of ICMT dominant negative form (ICMT-E167A) [19] (Figure 2G, right panel).

### 3.3. ICMT-Mediated Inflammatory Response Is Mediated by Ras

**Figure 3:**
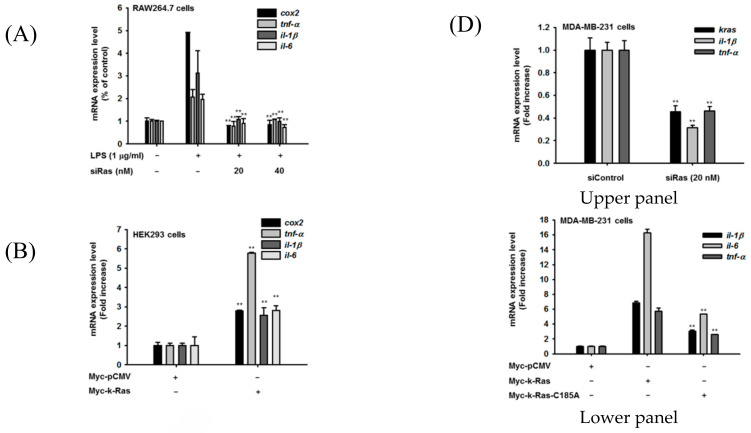

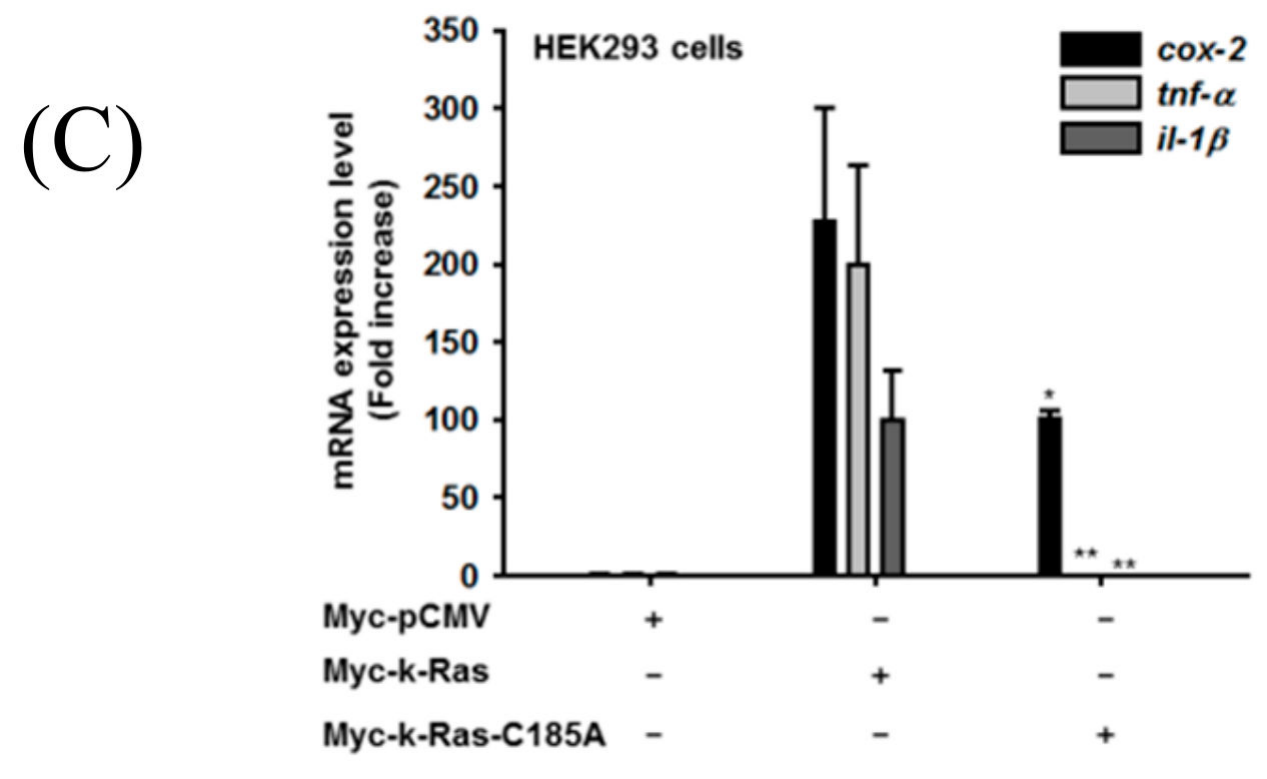
ICMT-mediated inflammatory responses are mediated by Ras. (**A**) The mRNA levels of inflammatory genes (*COX-2*, *TNF-α*, *IL-1β*, and *IL-6*) in RAW264.7 cells treated with 1 µg/mL of LPS in the presence or absence of siRas were determined by real-time PCR. (**B**,**C**) The mRNA levels of inflammatory genes (*COX-2*, *TNF-α*, *IL-1β*, and *IL-6*) were also determined in HEK293 cells transfected with Myc-k-Ras or Myc-k-Ras-C185A using real-time PCR. ((**D**), upper and lower panels) The mRNA levels of *K-RAS* and inflammatory genes (*IL-6*, *TNF-α*, and *IL-1β*) in MDA-MB-231 cells treated with siRNA to Ras (left panel) or transfected with Myc-k-Ras or Myc-k-Ras-C186A (right panel) were determined by real-time PCR. *: *p* < 0.05 and **: *p* < 0.01 compared to the normal group, LPS-treated group, or Myc-k-Ras-transfected group.

Since previous results indicate that ICMT could play a critical role in TLR-mediated inflammatory responses, we next tried to understand the mechanistic basis of this effect. For this, we focused on Ras, a well-known substrate of ICMT that is post-translationally modified by methylation in isoprenylated cysteine (Cys-185) [47], which was increased under the same conditions in vitro and in vivo (Figure 1). First, to confirm that LPS-activated Ras plays a critical role in ICMT-mediated inflammatory responses during TLR activation, we treated siRNA targeting *K-RAS* (siRas) to LPS-activated RAW264.7 cells. As Figure 3A shows, siRas treatment completely suppressed the expressions of *COX-2*, *TNF-α*, *IL-1β*, and *IL-6*. In contrast, *RAS*-overexpressing HEK293 cells also displayed increased levels of *COX-2*, *TNF-α*, *IL-1β*, and *IL-6* from 3- to 6-fold (Figure 3B). Interestingly, suppression of Ras methylation by overexpression of unmethylated Ras mutant (RAS-C185A, Figure S4) displayed weak expressions of those inflammatory genes (Figure 3C). In addition, we also examined whether Ras is able to control inflammatory responses in cancer cells. For this, we employed MDA-MB-231 breast cancer cells and assessed the levels of inflammatory genes under siRas treatment or Ras overexpression conditions. As seen in macrophages and HEK293 cells (Figure 3A–C), knockdown of *RAS* clearly suppressed the levels of *IL-1β* and *TNF-α* in breast tumor cells (Figure 3D, upper panel). In contrast, overexpression of *RAS* enhanced the expression levels of *IL-1β*, *IL-6*, and *TNF-α*, while the induction effect of unmethylated Ras was significantly reduced compared to wild type (Figure 3D, lower panel). 

### 3.4. ICMT-Methylated Ras-Mediated Inflammatory Response Is Managed by the MAPK-AP-1 Pathway

**Figure 4:**
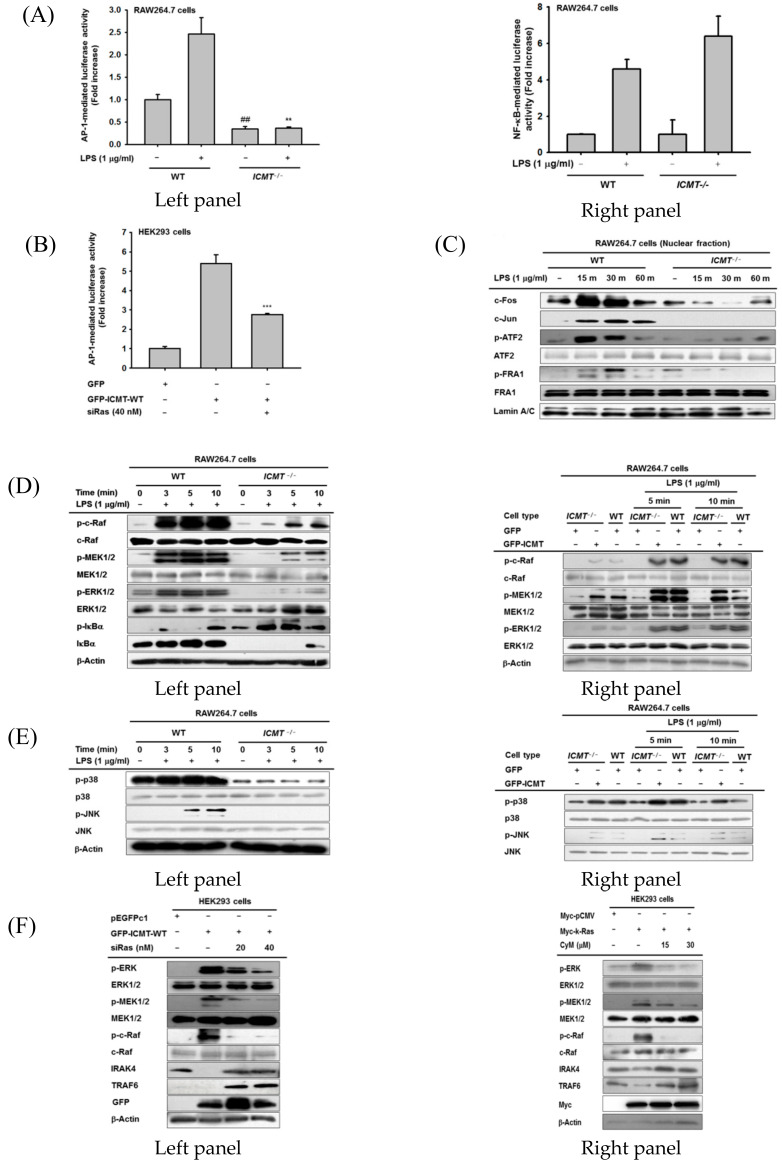

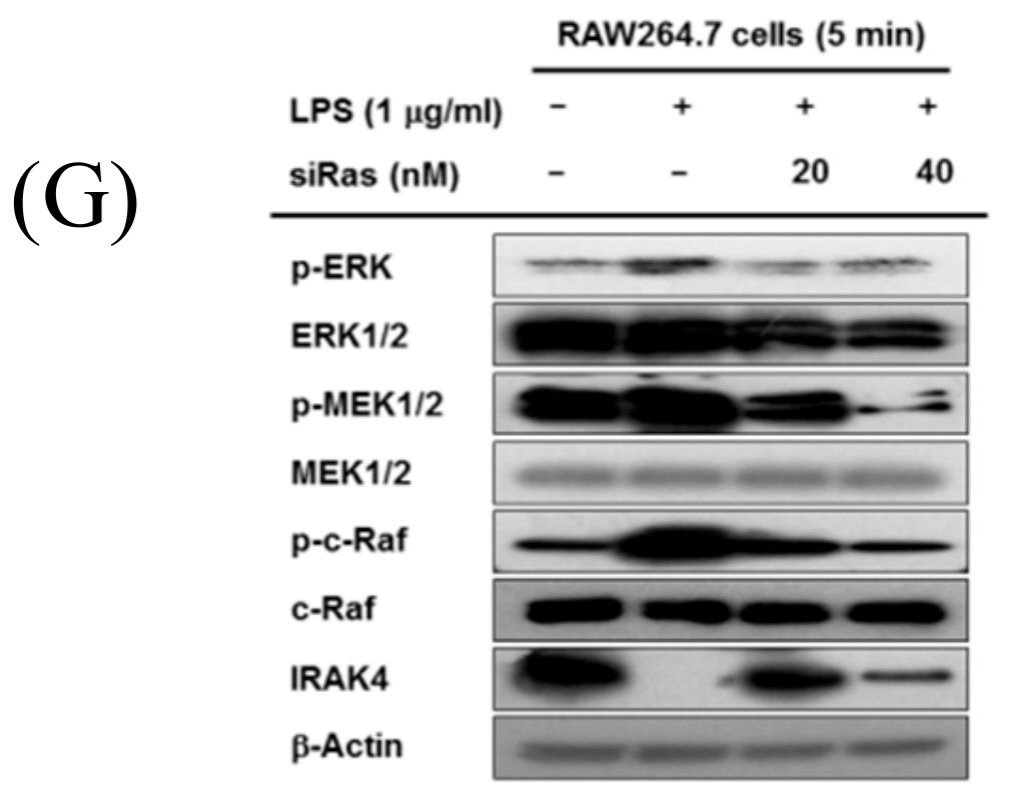
ICMT-mediated Ras activation reaches the AP-1 pathway. (**A**) AP-1 or NF-κB-mediated luciferase activity was analyzed by reporter gene assay in RAW264.7-WT and RAW264.7-*ICMT*^-/-^ cells treated with LPS (1 µg/mL). (**B**) AP-1-mediated luciferase activity was analyzed by reporter gene assay in HEK293 cells transfected with GFP-ICMT-WT (left panel) in the presence or absence of siRas. (**C**) Nuclear translocation of AP-1 subunits, including c-Fos, c-Jun, p-ATF-2, and p-FRA1, was identified by immunoblotting assay in nuclear fractions of RAW264.7-WT and RAW264.7-*ICMT*^-/-^ cells treated with LPS (1 µg/mL) for the indicated times. (**D**–**G**) The phospho- and total forms of c-Raf, MEK1/2, ERK1/2, IκBα, Src, p38, JNK1/2, and β-actin were measured by immunoblotting assay in RAW264.7-WT cells and in RAW264.7-*ICMT*^-/-^ cells treated with LPS (1 µg/mL) for 3 to 10 min ((**D**), left, and (**E**), left panels), in RAW264.7-WT cells and RAW264.7-*ICMT^-/-^* cells treated with with LPS (1 µg/mL) in the presence or absence of transfected *ICMT* ((**D**), right, and (**E**), right panels), in HEK293 cells transfected with GFP-ICMT and Myc-k-Ras in the presence or absence of siRas ((**F**), left panel) and CyM ((**F**), right panel), and in LPS-treated RAW264.7 cells in the presence or absence of siRas (**G**).

Because ICMT and its methylated protein K-Ras were found to play important roles in TLR-mediated expression of inflammatory genes, we sought to determine which transcription factors are activated by these molecules. As Figure 4A (left and right panels) shows, luciferase activity mediated by AP-1 but not NF-κB was abrogated under *ICMT*^-/-^ conditions. Moreover, overexpression of *ICMT* or *K-RAS* increased AP-1 activity, while this upregulation was suppressed by siRas (Figure 4B). In addition, there was no increase or suppression of luciferase activity triggered by NF-κB, CREB, STAT1 and STAT3 during overexpression of ICMT, Ras, MyD88 or TRIF in HEK293 cells in the presence or absence of CyM (Figure S5). However, the luciferase activity observed in IFN-γ-Promoter-Luc with binding sites for transcription factors (AP-1, NF-AF, AP-4, ATF, T-bet, and GATA) [49], was significantly reduced by CyM, implying that partial activity of AP-1could be affected by ICMT (Figure S5D). Therefore, we focused on the AP-1 pathway as a targeted transcription factor involved in ICMT/K-Ras-mediated inflammatory responses. To confirm the activity of AP-1, we also determined the nuclear translocation patterns of the AP-1 family, including c-Fos and AP-1 from *ICMT*^-/-^ cells. As expected, LPS strongly enhanced the translocation levels of c-Fos, c-Jun, phospho (p)-ATF2, and p-FRA1 at 15 and 30 min, whereas *ICMT*^-/-^ conditions remarkably suppressed the nuclear levels of these transcription factors at all time points (Figure 4C). We further analyzed the phosphorylation levels of MAPKs and their upstream kinases from these cells. Indeed, ICMT^-/-^ cells failed to show upregulation of p-ERK, p-p38, and p-JNK but not IκBα (Figure 4D,E, left panels). The phosphorylation of upstream kinases of ERK including c-Raf and MEK1/2 was significantly inhibited in *ICMT^-/-^* cells compared to control cells (Figure 4D, left and right panels). In addition, the overexpression of *ICMT* in *ICMT^-/-^* cells recovered the phosphorylation of c-Raf, MEK, ERK, p38, and JNK1/2 (Figure 4D,E, right panels), implying that the phosphorylation of MAPKs and their upstream proteins is managed by ICMT-derived molecular events. Since the functional role of ICMT was tightly linked to Ras (Figure 3D and Figure 4B), we next treated siRas under ICMT overexpression conditions or ICMT inhibitor CyM to K-RAS-overexpressed HEK293 cells to determine whether the MAPK activation pathway is also triggered by ICMT-methylated Ras. As we expected, siRas and CyM strongly suppressed the phosphorylations of ERK, MEK1/2, and c-Raf, and degradation of IRAK4 and TRAF6 induced by ICMT and K-RAS overexpression (Figure 4F), suggesting a critical role for methylated Ras in the activation of MAPKs and their upstream kinases. In addition, LPS/TLR4-induced activation of macrophages also increased the phosphorylations of ERK, MEK1/2, and c-Raf, and the degradation of IRAK4, whereas siRas strongly suppressed these events (Figure 4G).

### 3.5. ICMT-Methylated Ras-Mediated Inflammatory Responses Are Both MyD88- and TRIF-Dependent

**Figure 5:**
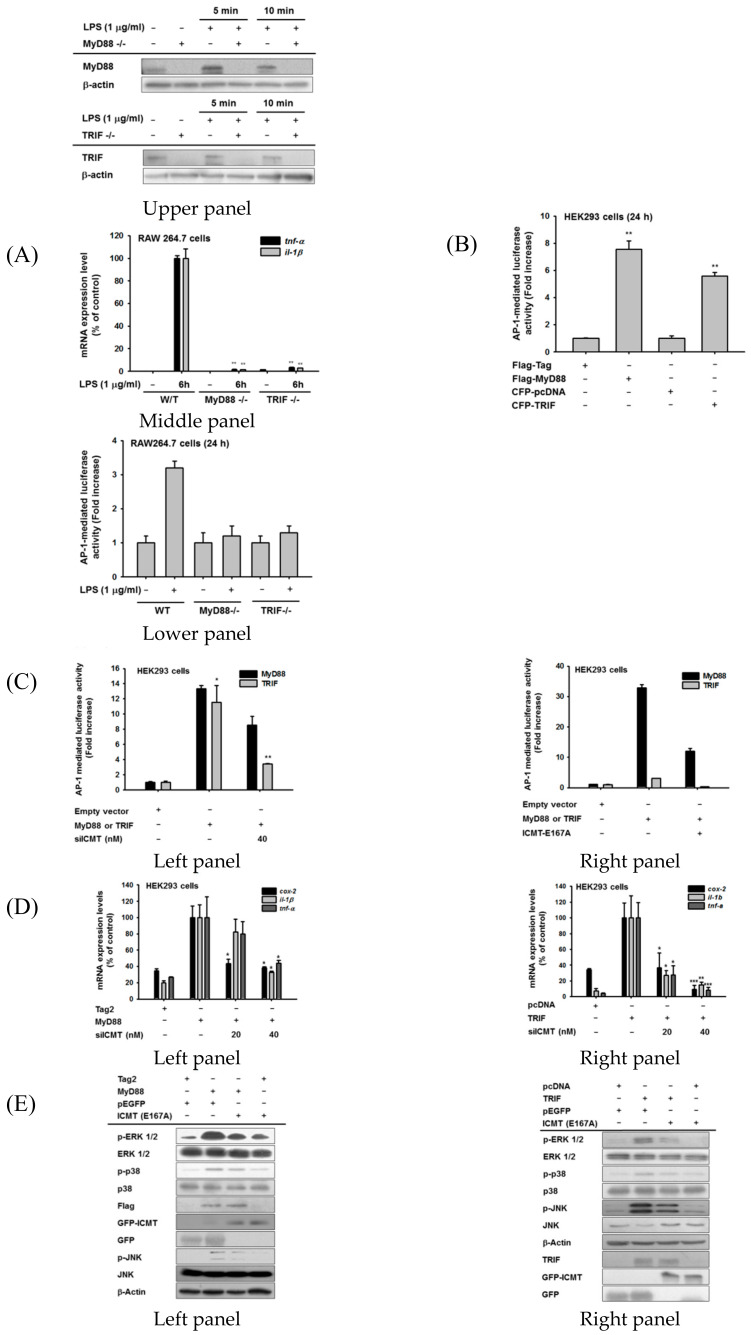

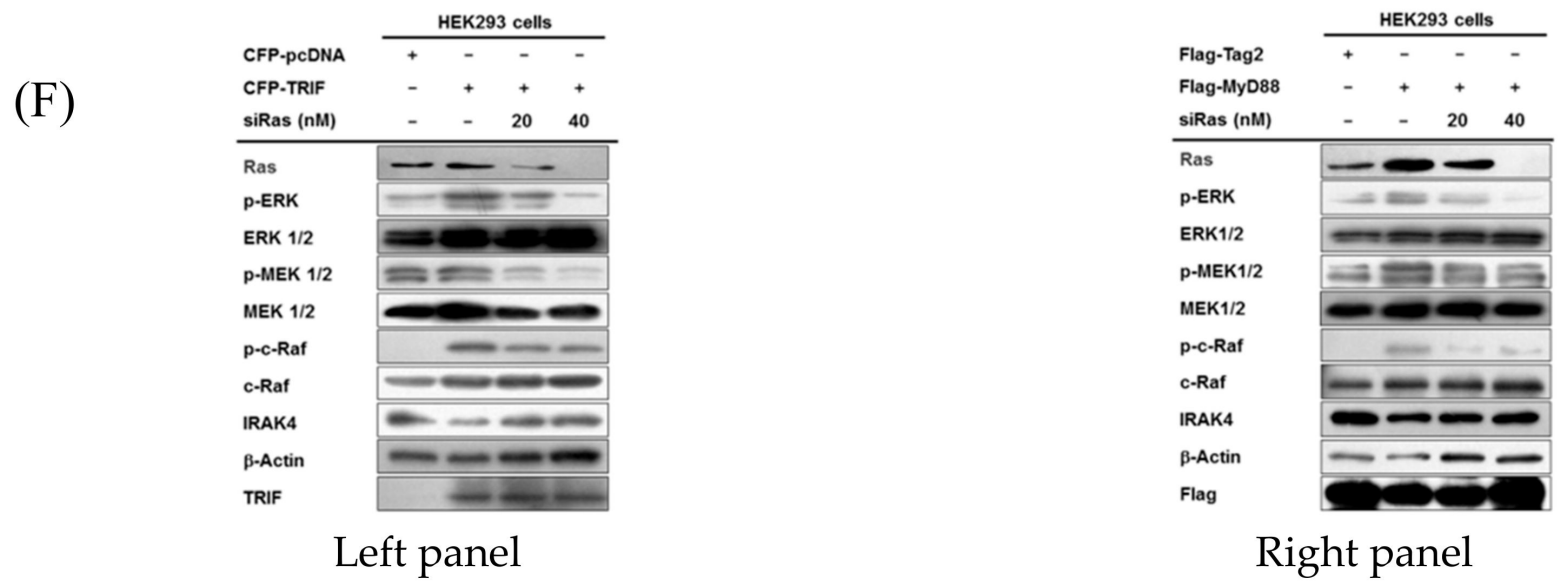
ICMT/Ras expression is both MyD88- and TRIF-dependent. ((**A**), upper panel) Protein levels of MyD88 and TRIF in RAW264.7 cells under their knockout conditions in the presence or absence of LPS (1 µg/mL). (**A**, middle panel) Inhibitory effects of *MyD88* and *TRIF* knockout on the expression of inflammatory genes (*TNF-α* and *IL-1β*) in RAW264.7 cells stimulated with LPS (1 µg/mL). ((**A**), lower panel, (**B**,**D**)) AP-1-mediated luciferase activity was analyzed by reporter gene assay in MyD88- and TRIF-knockout RAW264.7 cells ((**A**), lower panel), HEK293 cells transfected with Flag-MyD88 or CFP-TRIF (**B**), or HEK293 cells transfected with Flag-MyD88 or CFP-TRIF in the presence or absence of siICMT or ICMT-E167A ((**C**), left and right panels). Luminescence levels were determined with a luminometer. (**D**) mRNA expression levels of *COX-2*, *TNF-α*, *and IL-1β* were determined using real-time PCR in HEK293 cells transfected with MyD88 ((**D**), left panel) or TRIF ((**D**), right panel). (**E**) Total and phospho-protein levels of MAPK in HEK293 cells during transfection with *MyD88*, *TRIF*, and the dominant negative form of *ICMT* (*ICMT-E167A*). (**F**) Total and phospho-protein levels of ERK1/2 and their upstream enzymes (MEK1/2, c-Raf, and IRAK4) in HEK293 cells transfected with MyD88 or *TRIF* in the presence or absence of siRas were analyzed using immunoblotting analysis.

To determine which adaptor molecule is involved in ICMT-methylated/Ras-mediated induction of inflammatory gene expression, we prepared two sets of knockout cells: one without *MyD88* (RAW264.7-MyD88^-/-^ cells) and another without *TRIF* (RAW264.7-TRIF^-/-^ cells) (Figure 5A, upper panel). The expressions of *TNF-α* and *IL-1β* were strongly diminished in both sets of cells (Figure 5A, middle panel). Since ICMT and its methylated Ras mainly activate the AP-1 pathway, we next investigated the induction levels of AP-1 promoter activity in both RAW264.7-MyD88^-/-^ and -TRIF^-/-^ cells during LPS exposure using a reporter gene assay. As Figure 5A (lower panel) shows, there was no increase of AP-1-mediated luciferase activity in either type of knockout cell, implying that both molecules are important for AP-1 activation (Figure 5A, lower panel). Similarly, overexpressions of MyD88 and TRIF upregulated the promoter activity of AP-1 from 4- to 7-fold (Figure 5B), while such increases were suppressed by transfection with siICMT (Figure S6) and the dominant negative form of ICMT (ICMT-E167) (Figure 5C). Consistently, MyD88 and TRIF overexpression increased the expressions of *COX-2*, *IL-1β*, and *TNF-α*, whereas siICMT transfection significantly blocked the inductions of genes triggered by MyD88 and TRIF overexpression (Figure 5D, left and right panels). Since MyD88 and TRIF induced AP-1 activation and inflammatory gene expression in an ICMT-dependent manner, we next determined whether MyD88 or TRIF is able to trigger the AP-1 activation signaling pathway in an ICMT-dependent manner. Interestingly, the activations of ERK, p38, and JNK, as assessed by identification of their phosphorylation levels, were found to be upregulated by MyD88 and TRIF, while overexpression of ICMT-E167A strongly reduced the phosphorylation of MAPKs (Figure 5E). Similar effects were also observed under transfection of MyD88 and TRIF after siICMT transfection (Figure S7). We further confirmed whether Ras is involved in controlling ICMT-dependent MAPK activation induced by MyD88 or TRIF. Under knockdown conditions of Ras (Figure S8), the phosphorylations of ERK1/2, MEK1/2, and c-Raf, and degradation of IRAK4 triggered by MyD88 and TRIF were reduced by siRas treatment (Figure 5F, left and right panels), implying that AP-1 activation by MyD88 and TRIF is managed by ICMT-methylated Ras.

### 3.6. The TIR Domains of Both MyD88 and TRIF Play Functionally Important Roles in ICMT-Methylated Ras-Mediated Inflammatory Responses

**Figure 6:**
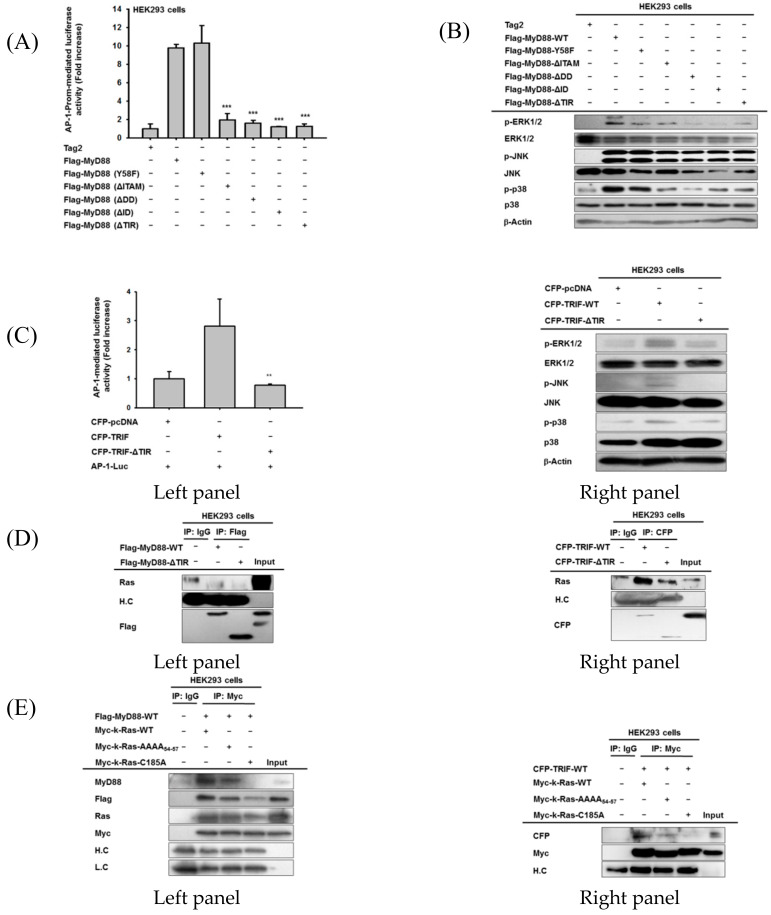

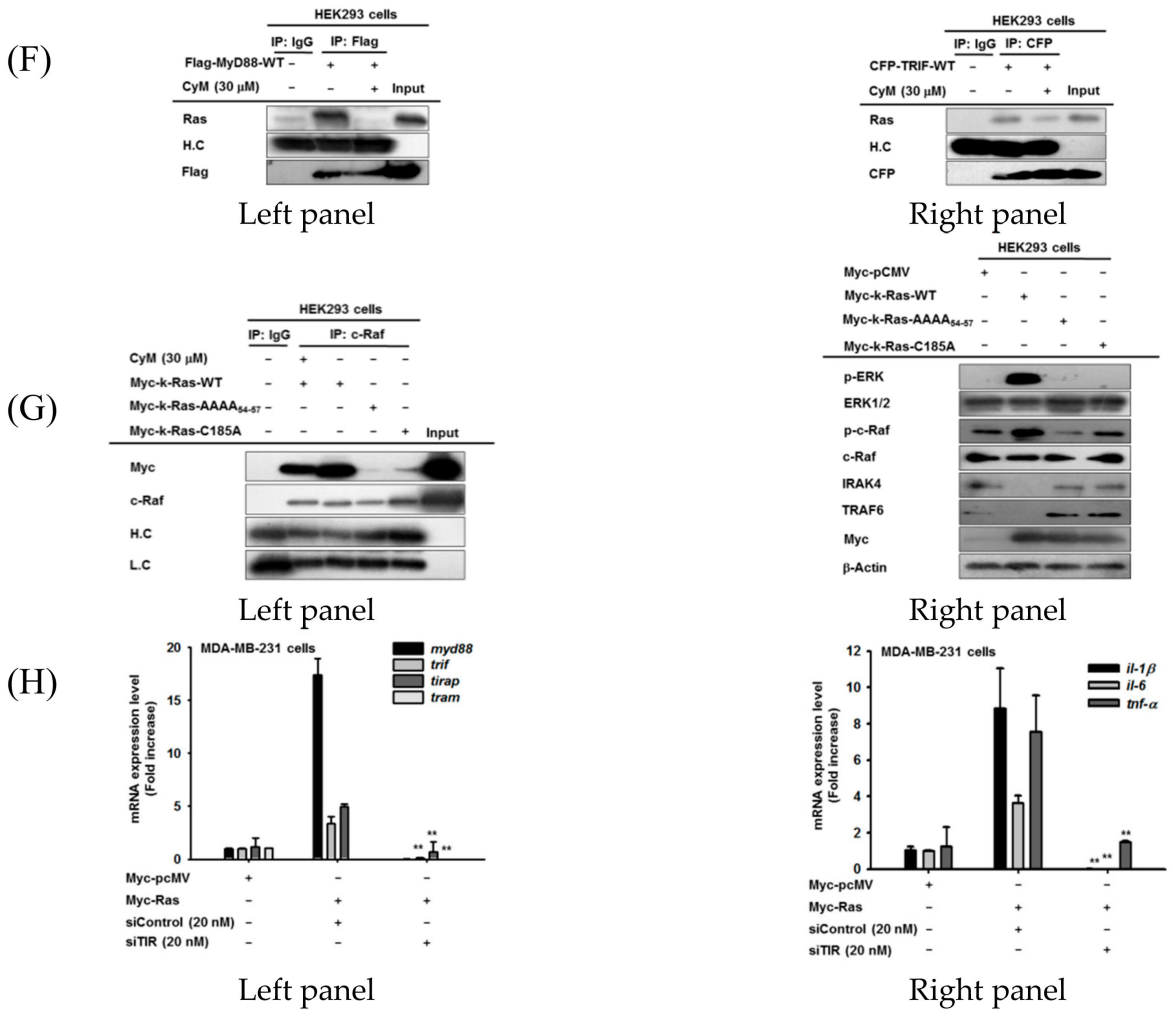
ICMT/Ras-mediated inflammatory responses are regulated by the TIR domain of MyD88 and TRIF. ((**A**,**C**), left panel) AP-1-mediated luciferase activity was analyzed by reporter gene assay in HEK293 cells transfected with *MyD88* and its mutant constructs [*MyD88* (*Y58F*), *MyD88* (Δ*ITAM*), *MyD88* (Δ*DD*), *MyD88* (Δ*ID*), and *MyD88* (Δ*TIR*)] (**A**) and in HEK293 cells transfected with *TRIF* and its mutant construct *CFP-TRIF-*Δ*TIR* ((**C**), left panel). Luminescence levels were determined with a luminometer. ((**B**,**C**), right panel) Total and phospho-protein levels of MAPKs (ERK1/2, JNK1/2, and p38) in HEK293 cells transfected with MyD88 and its mutant constructs [*MyD88* (*Y58F*), *MyD88* (Δ*ITAM*), *MyD88* (Δ*DD*), *MyD88* (Δ*ID*), and *MyD88* (Δ*TIR*)] (**B**) and in HEK293 cells transfected with *TRIF* and its mutant construct *CFP-TRIF-*Δ*TIR* ((**C**), right panel). ((**D**–**G**), left panel) Binding of endogenous Ras (**D**,**F**) and Myc-tagged *RAS* and its mutants (*Myc-K-RAS-AAA_54-57_* and *Myc-K-RAS-C185A*) (**E**) to MyD88 ((**D**,**E**), left panel), TRIF ((**D**,**E**), right panel), and c-Raf ((**G**), left panel) was evaluated using immunoprecipitation and immunoblotting analyses with total lysates of HEK293 cells transfected with *MyD88-WT* or *MyD88-*Δ*TIR* ((**D**,**E**), left panel) and *TRIF* and *TRIF-*Δ*TIR* ((**D**,**E**), right panel) in the absence (**D**,**E**) or presence ((**F**,**G**), left panel) of CyM (30 µM). ((**G**), right panel) Total and phospho-protein levels of ICMT, c-Raf, ERK1/2, IRAK4, and TRAF6 from whole lysates of HEK293 cells transfected with Myc-tagged *RAS* or its mutants (*Myc-K-RAS-AAA_54–57_* and *Myc-K-RAS-C185A*) were analyzed using immunoblotting analysis. (**H**) The mRNA expression levels of TIR-domains containing adaptor molecules (MyD88, TRIF, TIRAP, and TRAM) (**H**, left panel) and inflammatory genes (IL-1β, IL-6, and TNF-α) ((**H**), right panel) were measured using real-time PCR in MDA-MB-231 cells transfected with Ras in the presence or absence of siTIR (20 nM). **: *p* < 0.01 and ***: *p* < 0.001 compared to MyD88 or TRIF-WT-transfected group.

To determine the critical domains of MyD88 or TRIF that regulate AP-1 activation pathway, we next examined the promoter activity of AP-1 in several mutants of MyD88 or TRIF (Figure S9) using a reporter gene assay. Interestingly, as shown in Figure 6A, deletion of the death domain (DD), intermediate domain (ID), and TIR domains strongly affected the induction of AP-1 promoter activity up to basal levels. Similar to an AP-1-mediated luciferase activity assay (Figure 6A), molecular patterns of these mutants on the activation of MAPK, as assessed by phosphorylation level, were downregulated by transfection with deletion mutants without DD, ID, and TIR (Figure 6B). Since MyD88 and TRIF both contain TIR domains (Figure S9), we next evaluated the effects of the TIR domain of TRIF by preparing its TIR-deletion mutant on AP-1 activation and its related signalling pathway. Similar to MyD88-TIR domain deletion, transfection with CFP-TRIF-ΔTIR failed to increase AP-1-mediated luciferase activity (Figure 6C, left panel). The upregulated phosphorylations of ERK1/2, JNK, and p38 triggered by TRIF overexpression were not seen in CFP-TRIF-ΔTIR overexpression (Figure 6C, right panel). To further explore how the TIR domains of MyD88 and TRIF regulate the functional role of Ras for increasing ICMT-mediated inflammatory responses, we first performed an immunoprecipitation analysis. As shown in Figure 6D, Ras-binding capability to MyD88 or TRIF was clearly lowered in the MyD88 (left panel) and TRIF (right panel) mutants without TIR domains. To obtain more detailed information explaining how Ras recognizes the TIR domains of MyD88 or TRIF, we next mutated amino acids 54 to 57 of Ras, because they show similar polarity to the amino acids (208–211) of the TIR domain (Figure S4), and then we evaluated the ability of this mutant (Ras-AAAA_54–57_) to bind to MyD88 or TRIF. As shown in Figure 6E, the Ras mutant (Ras-AAAA_54–57_) failed to bind to MyD88 (left panel) and TRIF (right panel). Intriguingly, the binding activity of Ras to MyD88 or TRIF was shown to depend on ICMT activity; binding levels of MyD88 and TRIF to the unmethylated form of Ras (Ras-C185A) were markedly reduced (Figure 6E, left and right panels). In addition, treatment of cells overexpressing MyD88 or TRIF with CyM strongly reduced the binding of Ras to MyD88 (left panel) and TRIF (right panel) (Figure 6F). Consistent with this finding, overexpression of unmethylated Ras transfected with Ras-C185A or the TIR-unbound form of Ras transfected with Ras-AAAA_54–57_ suppressed the formation of its active form, as assessed by the binding level to c-Raf (Figure 6G, left panel). In agreement with this result, the overexpressions of Ras-AAAA_54–57_ and Ras-C185A did not increase the phosphorylation of c-Raf and ERK, or the degradation of IRAK4 and TRAF6 (Figure 6G, right panel). Finally, to confirm the significance of the TIR domain in Ras function, we designed siRNA for TIR domains (siTIR) of adaptor molecules, transfected it to Ras-overexpressing MDA-MB-231 cells, and determined the expression levels of TIR-domain-containing adaptor molecules as well as pro-inflammatory cytokines. Intriguingly, Ras overexpression strongly upregulated the gene expression levels of TIR-containing adaptor molecules and pro-inflammatory cytokines in MDA-MB-231 cells (Figure 6H). However, transfection of siTIR remarkably reduced the expression levels of TIR-domains containing adaptor molecules (*MYD88*, *TRIF*, and *TIRAP*), although expression of *TRAM* was not induced by Ras overexpression (Figure 6H, left panel). In agreement with this finding, siTIR strongly suppressed the expressions of pro-inflammatory cytokine genes (IL-1β, IL-6, and TNF-α) induced by Ras overexpression (Figure 6H, right panel), implying that TIR domain-containing proteins play a critical role in Ras-triggered inflammatory responses in macrophages and even in cancer cells. 

## 4. Discussion

In the present study, we demonstrated that the enzyme ICMT, which mediates Ras methylation, plays an important role in inflammation and related diseases. Previously, it was shown that ER trafficking and plasma membrane translocation of Ras are regulated by methylation [50], and that ERK1/2 and MEK1/2 are critical signaling proteins involved in activating the AP-1 pathway [51]. Therefore, we first focused on isoprenylcysteine methylation by ICMT in in vitro and in vivo inflammatory responses. Early clinical studies showed that the inhibition of Ras farnesylation or geranylation was not particularly effective due to compensation, suggesting that the final step of methylation mediated by ICMT could be a critical step for therapeutic targeting. Consistent with that hypothesis, the expressions of ICMT and Ras were increased in inflammatory diseases in both animal models and humans (Figure 1 and Figure S3). Moreover, our data (Figure 2 and Figure S6) suggest that inhibition of ICMT by knockout and knockdown markedly attenuates Ras-mediated inflammatory responses not only in vitro in macrophages and breast cancer cells, but also in vivo disease models. This suggests roles for ICMT and its methylated Ras in inflammatory responses managed by immune cells, making ICMT a good therapeutic target for future clinical applications regarding acute and chronic inflammatory diseases. 

**Figure 7:**
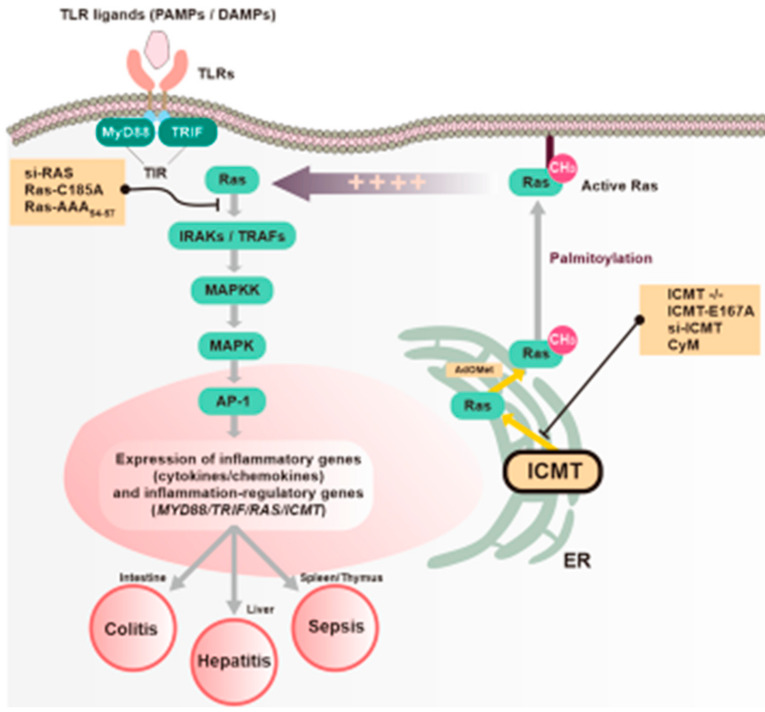
Proposed model of TIR domain-dependent regulation of Ras- and ICMT-mediated inflammatory responses in macrophages.

Several of our ICMT and Ras experimental findings are novel. First, the inflammatory response increases the expressions of ICMT and its methylated substrate Ras, and their cooperative activities via the AP-1 pathway in TLR-activated macrophages (Figure 1 and Figure 4). Previous studies of ICMT and Ras have been limited to cancer research [52]; this study is the first to examine its role in the context of inflammatory stimuli at molecular levels. Second, we demonstrated the roles of ICMT in Ras-dependent inflammation using a variety of approaches, in in vitro and in vivo models (Figure 4 and Figure 7), thereby confirming that ICMT is a viable clinical target for new therapeutics against various inflammatory symptoms. Third, ICMT regulates the IRAK-MAPKK-MAPK-AP-1 signaling system but not NF-κB and STATs (Figure 4 and Figure S5), which is the major signaling pathway associated with the inflammatory response, in a Ras-dependent manner. In previous study, there was no report explaining the relationship between NF-κB and ICMT or Ras, implying that ICMT-induced inflammatory signaling seems to be AP-1-dependent. In fact, it was obviously known that Ras/Raf/MEK/ERK and Ras/MEKK-1/JNKK-1/JNK are major canonical pathways to activate AP-1 in the cell system [53]. Suppression of ICMT by adenosine dialdehyde and siICMT was also found to reduce a role of AP-1 in breast tumor cells [51]. Thus, AP-1, and ICMT and its methylated Ras, could play complementary roles in the inflammatory response. Fourth, AP-1 activation signaling managed by ICMT-methylated Ras is simultaneously regulated by both MyD88 and TRIF via interactions with their TIR domains as well as other domains of MyD88 (Figure 5 and Figure 6). We found that ICMT-methylated Ras induces the expressions of TIR domain–containing proteins such as MyD88, and TRIF in macrophages (data not shown) and MDA-MB-231 cells, essential events in inflammatory responses (Figure 6H). Although the roles of Ras and ICMT in cell signaling were studied previously, the scope of those studies was narrow, and the exact mechanisms underlying inflammation were not identified. In this study, we explained the roles of ICMT and Ras in inflammation, and that they are managed by interactions with the TIR domains of adaptor molecules for AP-1 signaling activation. 

Ras is a small GTPase that regulates biological functions including cell survival, differentiation, and proliferation [54,55]. For Ras to be activated, the -AAX residues attached to the cysteine must be cleaved off by RCE1, after which Ras translocates to the ER and is methylated by ICMT [50]. K-Ras is involved in this process, whereas N-Ras and H-Ras are modified by palmitoylation in the Golgi [29,31,56,57]. We found that LPS increased the localization of Ras in the ER, which was suppressed by the ICMT inhibitor CyM, as observed by confocal microscopy (Figure 1D). Moreover, unmethylated Ras with an alanine at the Cys-185 residue showed defective activity in inducing inflammatory responses in HEK293 and MDA-MB-231 breast cancer cells (Figure 3C,D), and interacting with MyD88 and TRIF, implying that ICMT plays a critical role in Ras function, as reported previously for Ras-dependent signaling cascades [28]. mRNA expression of *K-RAS* appeared to be tightly regulated by TLR2, TLR3, and TLR4 stimulation (Figure 1F), which depended on both the MyD88 and TRIF adaptor molecules (data not shown), implying that Ras activation is essential in any circumstance, including G(−) and G(+) bacterial and viral infectious conditions. Under *ICMT^-^*^/-^ conditions in RAW264.7 cells, most Ras family members, including *K-RAS*, *H-RAS*, *N-RAS*, *RANPBP1*, *RHOC*, and *RASA1*, showed 50% or greater reductions in expression during LPS stimulation, implying that Ras and its related molecules are important in modulating inflammatory responses in immune and cancer cells mediated by PRRs or other stimuli including cytokines. It is well-known that damage-associated molecular patterns including HMGB1 and ATP can also activate macrophages via TLRs [58]. Thus, our findings suggest that sterile inflammatory events generated by physical damage to tissues or organs also activate Ras and its related damage-associate pattern receptors via TLR4. Indeed, the inflammatory disease models we established in this study indicate the importance of Ras in inflammatory diseases. For example, Ras was highly expressed in hepatitis induced by LPS/D-GalN, gastritis induced by HCl/EtOH, and colitis induced by DSS (Figure 1I). In agreement with our findings, several previous studies reported functional relationships between TLR4 and Ras-related proteins. For example, LPS treatment of cultured human cholangiocytes (NHC and H69 cells, the epithelial cells lining the bile ducts in the liver) strongly triggered the activation of N-Ras via TLR4/MyD88 [59,60]. A Ras-related brain protein, Rab10, also mediates LPS signaling for the induction of inflammatory gene expression. Reduction in Rab10 expression has been linked to suppression of TNF-α and IL-6 in LPS-treated RAW264.7 macrophages [61]. However, Ras guanine nucleotide-releasing protein 3 (RasGRP3), a type of GTP/GDP exchange factor responsible for the activation of Ras or Raps, is decreased in LPS-treated macrophages from 1 to 9 h [4], implying that the Ras-related pathway has both positive and negative roles in macrophage-mediated inflammatory responses. A protein farnesyltransferase inhibitor, tipifarnib, failed to suppress LPS-induced inflammatory responses, such as cytokine (IL-1β, IL-6, and TNF-α) and chemokine (MCP-1 and MIP-1α) expression [62], indicating that protein farnesyltransferase inhibition is not restricted to the Ras pathway. Although Ras-related proteins are also known to be inflammatory regulators, our data and previous research outcomes suggest that Ras proteins, including K-Ras and N-Ras, are important molecules in macrophage-mediated inflammatory responses also mediated by TLRs.

One interesting finding is that sustained and prolonged inflammation causes cancer [63]. Molecular events including secretion of toxic radicals (e.g., reactive nitrogen species and reactive oxygen species), production of cytokines that enhance tumor cell growth (including TNF-α, IL-6, and IL-10), upregulation of TGF-β (which increases tumor cell tissue invasion), and increase in the expression of IL-17 (which upregulates angiogenesis) [64,65,66] are some of the means by which inflammatory conditions are linked directly to tumorigenic responses. The molecular responses that result in syntheses of various cytokines and radical-inducing genes are mediated in part by AP-1 and its activating enzymes, including ERK, JNK, and p38 [67,68]. Our results indicate that Ras and ICMT are also key molecules in those events. Indeed, various acute and chronic inflammatory diseases, including gastritis, colitis, Crohn’s disease, ulcerative colitis, inflammatory bowel disease, and Hashimoto’s thyroiditis (data not shown), which are major causes of cancer in the colon, stomach, and thyroid, respectively, exhibited elevated protein and transcription levels of ICMT and Ras (Figure 1). Moreover, stable expression of the active form of Ras in the Chief cells of Mist1-CreERT2Tg/+ and LSL-K-Ras(G12D)Tg/+ (Mist1-Kras) mice, which express the active form of K-Ras upon exposure to tamoxifen, can cause gastric cancer with parietal cell loss, spasmolytic polypeptide-expressing metaplasia, and intestinal metaplasia with increased inflammatory responses [69]. Some infectious conditions also increase the incidence of cancer [65]. Thus, *Fusobacterium nucleatum*–induced proliferation of colorectal cancer cells and tumor development activate TLR4 and RASA1 signaling [70]. In addition, activation of TLR2 by endogenous extracellular matrix-derived proteoglycan versican and TLR4 by HMGB-1 from damaged cells are known to promote tumor growth in a context-dependent manner [63]. These findings imply that strong and prolonged expression and activation of Ras and ICMT, associated with TLRs, critically contribute to a variety of pathophysiological features, including cytokine gene expression and migration, invasion, and anti-apoptotic responses, in both immune and cancer cells. In fact, we also found that overexpressions of *ICMT* and *RAS* in MDA-MB-231 cells enhance the expressions of VEGF, TNF-α, TGF-β, and IL-1β (Figure 3D, data not shown), thereby supporting this hypothesis. Other oncogenic proteins, including Src and JAK, are also known be activated in both inflammatory and cancerous conditions [71,72,73]. Developing therapeutic strategies to selectively suppress proteins that are functionally important in both inflammation and cancer could prevent the pathological link between disorders, as suggested by a previous report [63,71]. We intend to test this hypothesis in in vivo models in future studies. 

Adaptor molecules play important roles in the inflammatory responses of the TLR system. The identification of specific domains that mediate binding and signaling is important for development of new therapeutics that can enhance or disrupt binding. This is also true in the case of TLRs, where identification of binding domains in the adaptor molecules that mediate intracellular signaling promote the development of new therapeutics that target TLR signaling. When TLRs recognize pathogen-associated molecular patterns, the TIR domain in MyD88 mediates binding and signals the DD via the ID; then the DD transmits the signal to IRAK1 and IRAK4 to amplify the inflammatory signal [74,75,76]. Upon activation of TRIF, the TIR domain activates TRAF3 to stimulate Type 1 IFN production or TRAF6 to stimulate the production of pro-inflammatory cytokines [77,78]. We demonstrated here that deletion of the TIR domain of either MyD88 or TRIF was sufficient to inhibit TLR-induced/AP-1-dependent expressions of pro-inflammatory cytokine genes as well as molecular interactions between adaptor molecules and isoprenylcysteine-carboxylmethylated Ras (Figure 6), implying that the TIR domains of both MyD88 and TRIF are essential for mediating inflammatory responses of Ras. These results underscore the importance of the TIR domain in ICMT and Ras activation, but we also examined how Ras influences the functional role of adaptor molecules in immune cells and breast cancer cells at the molecular level. Using deletion mutants and immunoprecipitation assays, we found that the amino acid residues between 54 to 57 of Ras, which show similar polarity to the amino acids in the TIR domain, are crucial for its association with the TIR domains of both MyD88 and TRIF (Figure 6D). In addition, to determine whether ICMT plays a functional role in the interaction between Ras and its adaptor molecules, we investigated whether Ras binds to TRIF and MyD88 when ICMT is inhibited by CyM treatment or Ras is unmethylated by transfection with a Ras mutant, Myc-k-Ras-C185A, under the same conditions. As expected, when ICMT was inhibited and Ras was not carboxymethylated, binding between Ras and the adaptor molecules decreased (Figure 6F). Moreover, generation of the active form of Ras and binding to its downstream substrate, c-Raf, were reduced when the isoprenylcysteine carboxymethylation of Ras and its binding capacity to TIR domain of MyD88 or TRIF were blocked (Figure 6G). Thus, the TIR domains from MyD88 and TRIF, in addition to interacting with Ras, might be essential to the Ras-mediated signaling cascade and inflammatory responses in immune cells such as macrophages and even cancer cells. It was previously reported that Ras pathway activation is maintained in the presence of MyD88 and linked to the induction of the DNA repair enzyme ERCC1, enabling efficient DNA repair mechanisms in cancer cells [79]. In addition, MyD88 is known to mediate optimal activation of the Ras/ ERK pathway by binding to ERK [80]. It has also been proposed that MyD88 influences Ras-related cytoplasmic ERK1/2 levels, and that MyD88-dependent signaling enhances the expressions of genes that could contribute to breast cancer progression and genes previously associated with poor outcomes in breast cancer patients [81]. These findings, together with our data, suggest a tight functional relationship between TIR domain-containing proteins and Ras/ICMT. Therefore, whether the TIR domain is critical for Ras activation and how the TIR domain activates Ras under isoprenylcysteine carboxymethylated conditions in response to ICMT should be further examined by evaluating both inflammatory and cancerous responses.

## 5. Conclusions

In conclusion, we found that the expression and activation of ICMT and RAS were strongly upregulated in TLR-activated macrophages, and in various mouse and human inflammatory conditions. Molecular and pharmacological blockades of ICMT and Ras suppressed inflammatory activity in vitro and in vivo via inhibition of the AP-1 pathway in both immune and cancer cells. We determined that both MyD88 and TRIF play critical roles in ICMT/Ras-dependent inflammatory responses. Further molecular dissection studies to determine which adaptor molecule domains are critical and which molecular complexes are important for activation of the MAPK-AP-1 pathway revealed the importance of the association between ICMT-methylated Ras and the TIR domains of MyD88 and TRIF, as summarized in Figure 7. These results strongly suggest that ICMT/Ras are key proteins in the regulation of AP-1-mediated inflammatory responses managed by TIR-containing MyD88 and TRIF. Thus, ICMT-mediated methylation of Ras could serve as novel anti-inflammatory target reaction for inflammation-related diseases. Finally, since functional role of MyD88 or TRIF in inflammatory responses is cell-type specific in DSS-induced colitis and colorectal cancer models [82,83], therefore, we will also dissect functional involvement of ICMT/Ras pathway with different cells (e.g., intestinal epithelial cells) with knockout conditions of MyD88 and TRIF in the following projects.

## Figures and Tables

**Table 1:** siRNA sequences used in this study.

Target Protein or Domain	siRNA Sequence	
ICMT (human)	Sense	5′-CCAUAGCUUAUAUUCUCA-3′
	Antisense	5′-UUGAGAAUAUAAGCUAUGG-3′
ICMT (mouse)	Sense	5′-GCUACCAGAUAGCCAUCAG-3′
	Antisense	5′-CUGAUGGCUAUCUGGUAGC-3′
RAS (human)	Sense	5′-GUUGGAGCUGGUGGCGUAG-3′
	Antisense	5′-CTACGCCACCAGCTCCA-3′
RAS (mouse)	Sense	5′-GUGCAAUGAAGGGACCAGUA-3′
	Antisense	5′-UACUGGUCCCUCAUUGCAC-3′
TIR (human)	Sense	5′-GCGCGCGGAUGAACAUAUUUU-3′
	Antisense	5′-AAUAUGUUCAUCCGCGCGCUU-3′
Control (human)	Sense	5′-CCUACGCCACCAAUUUCGU-3′
	Antisense	5′-ACGAAAUUGGUGGCGUAGG-3′
Control (mouse)	Sense	5′-UUCUCCGAACGUGUCACGU-3′
	Antisense	5′-ACGUGACACGUUCGGAGAA-3′

**Table 2:** Primer sequences used in real time PCR analysis.

Targets	Sequence (5′ to 3′)	
*aoc3* (*Mus musculus*)	F	CAGCTCGGGACAGTGAGATA
	R	CCAGGTCTCAGCAAAGACAA
*aox1* (*Mus musculus*)	F	CAACCTTCCATCCAACACTG
	R	CCACATTTGATTGCCACTTC
*acox-2* (*Mus musculus*)	F	CACTACATCCTGACCCACTT
	R	ATGCTCCTGCTTGAGTATGT
*cox-2* (*Homo sapiens*)	F	AGGAGGTCTTTGGTCTGGTG
	R	TAGCCTGCTTGTCTGGAACA
*gapdh* (*Mus musculus*)	F	CAATGAATACGGCTACAGCAAC
	R	AGGGAGATGCTCAGTGTTGG
*gapdh* (*Homo sapiens*)	F	CGGGAAACTGTGGCGTGATG
	R	ATGACCTTGCCCACAGCCTT
*ho-1* (*Mus musculus*)	F	CAGAGAGTCAGCATGCCAAT
	R	ACTCAGCATCATGCCAGTTC
*h-ras* (*Mus musculus*)	F	GCCATCAACAACACCAAGTC
	R	GAATCTTTCACCCGCTTGAT
*icmt* (*Mus musculus*)	F	GATGGTGGTCTTCGGAGAAT
	R	CCACATGGTTGAAGTTGGAG
*icmt* (*Homo sapiens*)	F	GGTGACCAGTGGAGTGTACG
	R	GGGTTACACAGCATCACCTG
*il-1β* (*Mus musculus*)	F	GGCCTTGGGCCTCAAAGGAA
	R	GCTTGGGATCCACACTCTCCA
*il-1β* (*Homo sapiens*)	F	TGGACCTCTGCCCTCTGGAT
	R	AAGGTCTGTGGGCAGGGAAC
*il-4* (*Mus musculus*)	F	GCCATATCCACGGATGCAACG
	R	TTGCTGTGAGGACGTTTGGC
*il-6* (*Mus musculus*)	F	GACAAAGCCAGAGTCCTTCAGAGA
	R	CTAGGTTTGCCGAGTAGATCTC
*il-6* (*Homo sapiens*)	F	AAGCCAGAGCTGTGCAGATG
	R	CCTTGGTCACCGACGTCCTGT
*Inos* (*Mus musculus*)	F	GGAGCCTTTAGACCTCAACAGA
	R	TGAACGAGGAGGGTGGTG
*k-ras* (*Mus musculus*)	F	CAGGTGTTGAGGAGACCAGA
	R	CAGTAGCGTTCGTCACAGGT
*k-ras* (*Homo sapiens*)	F	TAGGCAAGAGTGCCTTGACG
	R	CCCTCCCCAGTCCTCATGTA
*myd88* (*Homo sapiens*)	F	CAGCTCTGAGCCATTCACAC
	R	CCAGCATGTAGTCCAGCAAC
*n-ras* (*Mus musculus*)	F	CAGAACGTGTGAGCTTGGTT
	R	GAACGCACACAGCTTCTGAT
*rasa1* (*Mus musculus*)	F	TCAGGTAGCAGCCTGTGTTC
	R	CTGGAATGTGGGAAAGTGTG
*ranbp1* (*Mus musculus*)	F	CCTGATGCCTTCCTTGTAGG
	R	AAAGCCCTTTCACTGCTGTT
*rhoc* (*Mus musculus*)	F	CCTGAGGCAAGATGAGCATA
	R	AGGTAGCCAAAGGCACTGAT
*tirap* (*Homo sapiens*)	F	TTCACAGCCTACCTCACAGG
	R	CACCAGGTCTTCCTCACTGT
*tnf-α* (*Mus musculus*)	F	TGCCTATGTCTCAGCCTCTT
	R	GAGGCCATTTGGGAACTTCT
*tnf-α* (*Homo sapiens*)	F	GGCCCAGGCAGTCAGATCAT
	R	TCTCTCAGCTCCACGCCATT
*tram* (*Homo sapiens*)	F	TCAGAGCGTGGAAGAGATGT
	R	CCACATGGCATCTCAGCAAA
*trif* (*Homo sapiens*)	F	CCATGATGAGCAACCTCACG
	R	ATCTGGGAGTGTTCGTCCAG

## Supplementary Materials

The following are available online at https://www.mdpi.com/2073-4409/9/5/1216/s1, Figures S1–S8, Tables S1–S2.
