# Supplementary material for: Isoprenylcysteine Carboxyl Methyltransferase and Its Substrate Ras Are Critical Players Regulating TLR-Mediated Inflammatory Responses
Source: Cells. 2020 May 14;9(5):1216. doi: 10.3390/cells9051216 (PMC7291029; doi:10.3390/cells9051216)
Supplement: Supplementary file 1 [file cells-09-01216-s001.pdf]

Supplementary information

Isoprenylcysteine carboxyl methyltransferase and its substrate Ras are critical players regulating TLR-mediated inflammatory responses

Woo Seok Yang [1,a], Han Gyung Kim [1,a], Eunji Kim[1,a], Sang Yun Han [1], Nur Aziz [1], Young-Su Yi [2], Sunggyu Kim [3], Yunmi Lee [4], Byong Chul Yoo [5], Jeung-Whan Han [6], Narayanan Parameswaran [7], Ji Hye Kim [1,*], and Jae Youl Cho [1,+]

[1] Department of Genetic Engineering, Sungkyunkwan University, Suwon 16419, Republic of Korea; real0902@gmail.com (W.S.Y.), hanks523@skku.edu (H.G.K.), im144069@gmail.com (E.K.), dangsukr@naver.com (S.Y.H.), nuraziz.20@gmail.com (N.A.), kjhmlkjhml@hanmail.net (J.H.K.) and jaecho@skku.edu (J.Y.C.) .

[2] Department of Life Science, Kyonggi University, Suwon 16227, Republic of Korea; ysyi@kgu.ac.kr (Y.S.Y.)

[3] Research and Business Foundation, Sungkyunkwan University, Suwon 16419, Republic of Korea; sukim590@skku.edu (S.K.K.)

[4] Department of Chemistry, Kwangwoon University, *Seoul* 01897, Republic of Korea; ymlee@kw.ac.kr (Y.L.)

[5] Division of Translational Science, Research Institute, National Cancer Center, Goyang 10408, Republic of Korea; yoo_akh@ncc.re.kr (B.C.Y.)

[6] Research Center for Epigenome Regulation, School of Pharmacy, Sungkyunkwan University, Suwon 16419, Republic of Korea; jhhan551@skku.edu (J.H.H.).

[7] Department of Physiology and Division of Pathology, Michigan State University, East Lansing, MI 48824, USA; narap@msu.edu (N.P.).

[+]Corresponding author. Email: jaecho@skku.edu and kjhmlkjhml@hanmail.net

[a]These authors equally contributed to this work

Short title: TIR domain-dependent inflammatory responses by ICMT and Ras

MATERIALS AND METHODS

Mice

Six-week-old ICR, Balb/c, and C57BL/6 male mice (see Table S1 for genetic background information) were purchased from Daehan Biolink (DBL, Chungbuk, Korea) and housed eight mice per group under a 12-h light/dark cycle (lights on at 6 a.m.).

Table S1. Genetic information of mouse used in this study.

Mouse (Nomenclature)	Company	Disease model	Genetic background
ICR (IcrRac:ICR)	DBL (Korea)	Gastritis	DBL received breeder stock from Taconic in 2010. Taconic received breeder stock from Fox Chase in 1993. Taconic's outbred colony is known to carry the recessive mutation, Pde6brd1
Balb/C (BALB/cAnNTac)	DBL (Korea)	Hepatitis	DBL received breeder stock from Taconic in 2010. Foundation colony of Taconic is F239 in 2009.<br>Strain Profile: Acy1s, Alada, Carb, Cas1a, Cd5b, Cd72b, Cd8ab, Cd8b1b, Ce2a, Es1b, Esda, pi1a, Gusa, H6pdb, Hbab, Hbbd, Hc1, Idh1a, Mod1a, Mup1a, Pepca, Pgm1a, Thy1b, Trfb, Ptprcb<br>Immunology: H2-T18c, H2d
C57BL/6 (C57BL/6NTac)	DBL (Korea)	Colitis	DBL received breeder stock from Taconic in 2014. Foundation colony of Taconic US is F1168 in 2009.<br>Strain Profile: Acy1f, Aladb, Car2a, Cas1g, Cd5b, Cd72b, Cd8ab, Cd8b1b, Ce2a, Es1a, Es3a, Esda, Ggc1b, Gpi1b, Gus-sb, H6pd1b, Hbaa, Hbbs, Hc1, Idh1a, Mod1b, Mup1b, Pep3a, Pgm1a, Trfb<br>Immunology: H2b, Ptprcb, Thy1b, H2-T18b<br>Retinal Degeneration: Crb1Rd8
NC/Nga (NC/NgaTndCrlj)	DBL (Korea)	Dermatitis	DBL received mice from Charles River Japan (CRJ). NC/Nga was established by Dr. Kondo et al of Nagoya University Japan in 1957. The colony maintained at Tokyo University of Agriculture and Technology was introduced to Charles River Japan in 1997. Genetic background of NC/Nga was published in [39].

Antibodies

Antibodies were purchased from Cell Signaling Technology (Beverly, MA, USA), Santa Cruz Biotechnology (Santa Cruz, CA, USA), and MERCK (Kenilworth, NJ, USA) (Table S2).

Table S2. List of antibodies used in this study and their catalog numbers

**Cell Signaling Technology**	
Antibody	Catalog Number
Anti-phospho ERK1/2	CST 4695
Anti ERK1/2 (Thr202/Tyr204)	CST 9101
Anti-SAPK/JNK	CST 9252
Anti-phospho-SAPK/JNK (Thr183/ Tyr 185)	CST 4668
Anti-p38	CST 9212
Anti-phospho-p38 (Thr180/Tyr182)	CST 9215
Anti-MEK1/2	CST 9122
Anti-phospho-MEK1/2 (Ser217/221)	CST 9121
Anti-MKK3	CST 9238
Anti-phospho-MKK3 (Ser189)/MKK6 (Ser207)	CST 9236
Anti-SEK1/MKK4	CST 9152
Anti-phospho-SEK1/MKK4 (Thr261)	CST 9151
Anti-c-Raf	CST 9422
Anti-phospho-c-Raf (Ser338)	CST 9427
Anti-MyD88	CST 3699
Anti-TRIF	CST 4596
Anti-DYKDDDDK	CST 2368
Anti-c-Jun	CST 9165
Anti-ATF2	CST 9226
Anti-phospho-ATF-2 (Thr71)	CST 9221
Anti-FRA 1	CST 5281
Anti-phospho-FRA1 (Ser265)	CST 3880
Anti-Lamin A/C	CST 4777
Anti-c-Fos	CST 2250
Anti-β-Actin	CST 4967
**Santa Cruz Biotechnology**	
Anti-ICMT	sc-161722
Anti-GFP	sc-9996
**Merck Millipore**	
Anti-RAS	05-516

Preparation of cell lysates and nuclear fractions from cells/tissues for immunoblotting analyses

Whole lysates were extracted from animal tissues, RAW264.7 cells, and HEK293 cells. Cells and tissues were washed with PBS, lysis buffer (20 mM Tris-HCl [pH 7.4], 2 mM ethylenediaminetetraacetic acid (EDTA), 2 mM ethyleneglycotetraacetic acid, 50 mM β-glycerophosphate, 1 mM sodium orthovanadate, 1 mM dithiothreitol [DTT], 1% Triton X-100, 10% glycerol, 10 μg/ml aprotinin, 10 μg/ml pepstatin, 1 mM benzamide, and 2 mM phenylmethanesulfonyl fluoride [PMSF]) was added, and samples were lysed at -70°C for 30 minutes. Lysates were clarified by centrifugation at 16,000 rpm for 10 min at 4°C and then stored at -20°C until needed. Extractions of nuclear and membrane fractions proceeded in a similar manner. Tissues or cells were washed with 400 μl of lysis solution A (50 mM KCl, 2 mM $MgCl_2$, 10 mM HEPES [pH 7.8] with KOH, 0.1 mM EDTA, 0.1 mM PMSF, 2 μg/ml leupeptin, 2 μg/ml aprotinin, and 1 mM DTT) and mixed vigorously for 30 seconds with 25 μl of 10% Nonidet P-40. After centrifugation at 14,000 RPM for 30 seconds, the supernatant (cytosolic fraction) and pellet (nuclear fraction) were separated. The supernatant was centrifuged at 14,000 rpm for 1 hour to obtain a membrane fraction. To isolate the nuclear fraction, we added 50 μl of lysis solution B (50 mM KCl, 300 mM NaCl, 10 mM HEPES [pH 7.8] with KOH, 0.1 mM EDTA, 0.1 mM phenylmethylsulfonyl fluoride, 2 μg/ml leupeptin, 2 μg/ml aprotinin, and 1 mM DTT) to the pellet and incubated it at 4°C for 20 minutes. During this time, vortexing was performed every 5 minutes. We then centrifuged the sample at 14,000 rpm for 5 minutes and collected the nuclear fraction. Proteins were separated by electrophoresis on 10–15% SDS-polyacrylamide gels and transferred to a polyvinylidene difluoride membrane by electroblot. The membrane was blocked by 3% bovine serum albumin (BSA) and dissolved in TBST (Tris-buffered saline containing 3% FBS, 20 mM NaF, 2 mM EDTA, and 0.2% Tween 20), and primary and secondary antibodies were then added. We determined the total and phosphorylated levels of inflammatory proteins using the ECL system (Amersham, Little Chalfont, Buckinghamshire, UK).

Human tissue data

Raw data from inflammatory diseases were assessed from the NCBI Gene Expression Omnibus using the following accession IDs: inflammatory cardiomyopathy, 26583460; rheumatoid arthritis, synovial tissues, 21212124; Crohn’s disease and ulcerative colitis comparison, 12532971; inflammatory bowel disease, 4225238; chronic obstructive pulmonary disease, 1638739; and alcoholic hepatitis, 85451358. Informed written consent was obtained from all participants.

Determination of PGE_2 production

We pre-incubated RAW264.7 cells (1×10^6 cells/ml) for 18 h, added ICMT-Ph (50 or 100 μM) for 1 h, and then further incubated them with LPS (1 μg/ml) for 24 h. The inhibitory effects of ICMT-Ph on the production of PGE_2 were determined using EIA kits (ThermoFisher Scientific), as described previously (Cho et al., 2000; Kim et al., 2013a).

Cell viability assay

To determine cell viability in the presence of ICMT inhibitors, we used the MTT assay as reported previoulsy. After pre-incubation of RAW264.7 cells, peritoneal macrophages, and HEK293 cells (1×10^6 cells/ml) for 18 h, ICMT inhibitors (CyM from 20 to 40 μM, ICMT-Ph from 1.5 to 200 μM) were added to the cells, which were then incubated for 24 h. The cytotoxicity of the ICMT inhibitors was evaluated using a conventional MTT assay, as described previously (Pauwels et al., 1988; Yayeh et al., 2012). Three hours prior to culture termination, 10 μl of MTT solution (10 mg/ml in PBS, pH 7.4) was added. The incubation was halted by the addition of 15% sodium dodecyl sulfate (SDS) to each well to solubilize the

formazan. The absorbance at 570 nm ($OD_{570-630}$) was measured using a Spectramax 250 microplate reader (BioTex, Bad Friedrichshall, Germany).

Immunoprecipitation assays

We performed immunoprecipitation assays to determine the binding activities of proteins. First, proteins were extracted using lysis buffer (20 mM Tris-HCl [pH 7.4], 2 mM EDTA, 2 mM ethyleneglycotetraacetic acid, 50 mM β-glycerophosphate, 1 mM sodium orthovanadate, 1 mM DTT, 1% Triton X-100, 10% glycerol, 10 μg/ml aprotinin, 10 μg/ml pepstatin, 1 mM benzamide, and 2 mM PMSF). For immunoprecipitation, we added 5 μl of the primary antibody and 1 μl of the control IgG to each sample group and incubated this mixture at 4°C for 24 hours. We then added protein A-coupled Sepharose beads (50% v/v) (Amersham, Little Chalfont, Buckinghamshire, UK) to the sample and allowed the proteins to bind to the beads in a rotating roller for 4 hours. After five washes with lysis buffer, we performed immunoblotting in the manner described above.

Confocal microscopy

We placed RAW264.7 cells plated at 7×10^5/ml in a 12-well plate and treated the cells 18 h later. After washing the cells three times for 5 min with 1 ml of PBS, we fixed the cells in 1 ml of 3.7% formaldehyde for 10 min. After washing, we permeabilized the cells for 5 min with 500 μl of 1% Triton x-100. After washing, 800 μl of 1% BSA was added, and the sample was blocked for 30 min. We next added the primary antibody (anti-ICMT or Ras) and then the secondary antibody (FITC-antibody: BD Sciences; ER tracker-Red: Invitrogen; Hoechst 33258: Sigma Chemical Co.). After washing, 20 μl of mounting solution (ThermoFisher Scientific) was applied, and the cover glass was placed on the slide, which was then analyzed using confocal microscopy (ZEISS LSM 800).

Microarray analysis

After preparing total RNA from RAW264.7 cells treated and untreated with LPS and from RAW264.7-*ICMT*$^{-/-}$ cells treated and untreated with LPS, we used the GeneChip® Mouse Gene 2.0 ST Array as a platform. cDNA was synthesized using the GeneChip Whole Transcript (WT) Amplification kit. The label protocol was as follows. First, sense cDNA was fragmented, and then biotin labeling of terminal deoxynucleotidyl transferase was performed using the GeneChip WT Terminal Labeling kit. Labeled target DNA was hybridized to the Affymetrix GeneChip Array at 45°C for 16 hours. Hybridized samples were washed, stained with a GeneChip Fluidics Station 450, and scanned with a GCS3000 scanner. Array data were processed using Affymetrix® GeneChip Command Console® software. Analyses were performed using Affymetrix® Expression Console™ software (http://www.affymetrix.com/estore/catalog/131414/AFFY/Expression-Console-Software#1_1) and R 3.1.2 (www.r-project.org). Except for total RNA preparation and data analysis, this process was performed by Macrogen, Inc. (Seoul, Korea).

Statistical analyses

All data presented in this paper are expressed as mean ± SD. For statistical comparisons, results were analyzed using either ANOVA/Scheffe's *post hoc* test or the Kruskal-Wallis/Mann-Whitney test. A *p*-value < 0.05 was considered to be statistically significant. All statistical tests were carried out using SPSS (SPSS Inc., Chicago, IL, USA). Similar experimental data were also obtained using an additional independent set of *in vivo* experiments conducted using the same numbers of mice.

Supplementary Figures

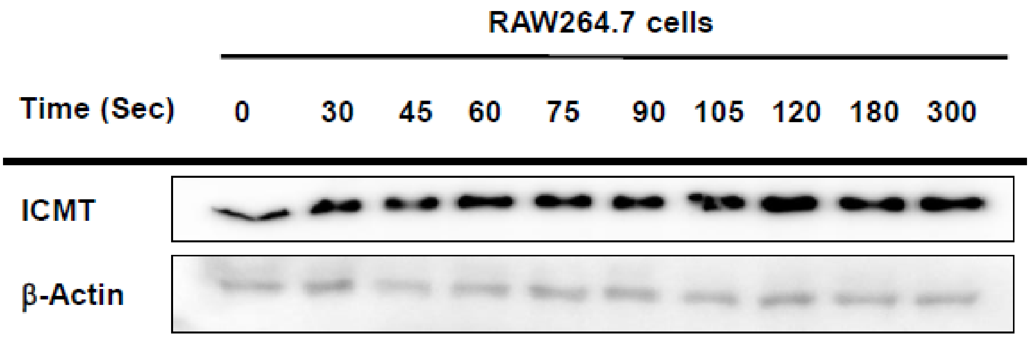

Figure S1. Protein level of ICMT under normal culture conditions of RAW264.7 cells**.** Protein level of ICMT in RAW264.7 cells was determined by immunoblotting analysis.

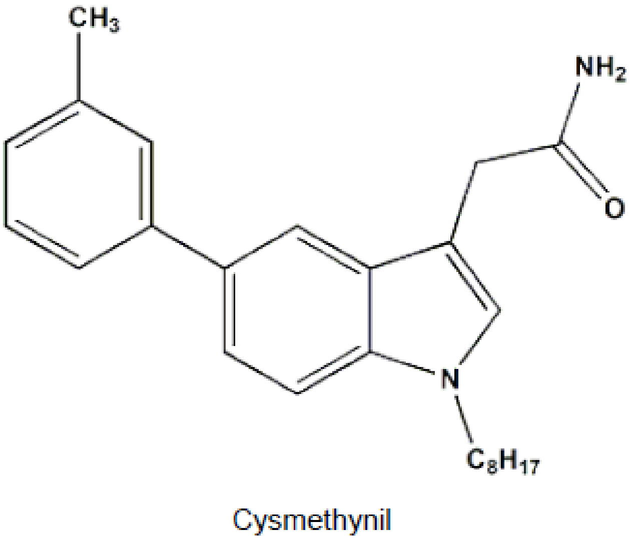

Figure S2. Chemical structure of cysmethynil (CyM).

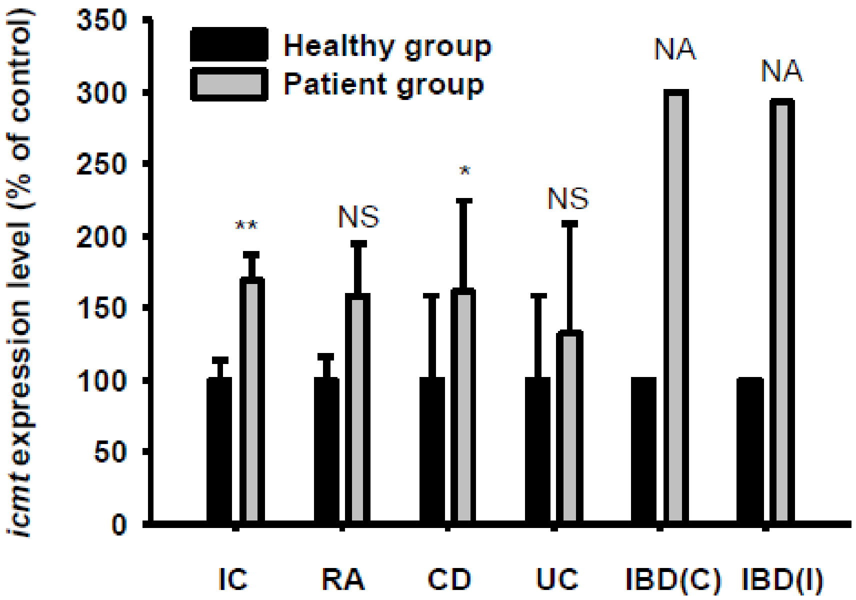

Figure S3. The mRNA expression level of ICMT in various disease conditions**.** mRNA expression level of *ICMT* in various human chronic inflammatory diseases was calculated using data from the NCBI Gene Expression Omnibus. *: $p < 0.05$ and **: $p < 0.01$ compared to healthy group. NS : not significant, NA : not available, IBD (C) : Inflammatory bowel diseases (Colon), and IBD (I) : Inflammatory bowel diseases (Ileum)

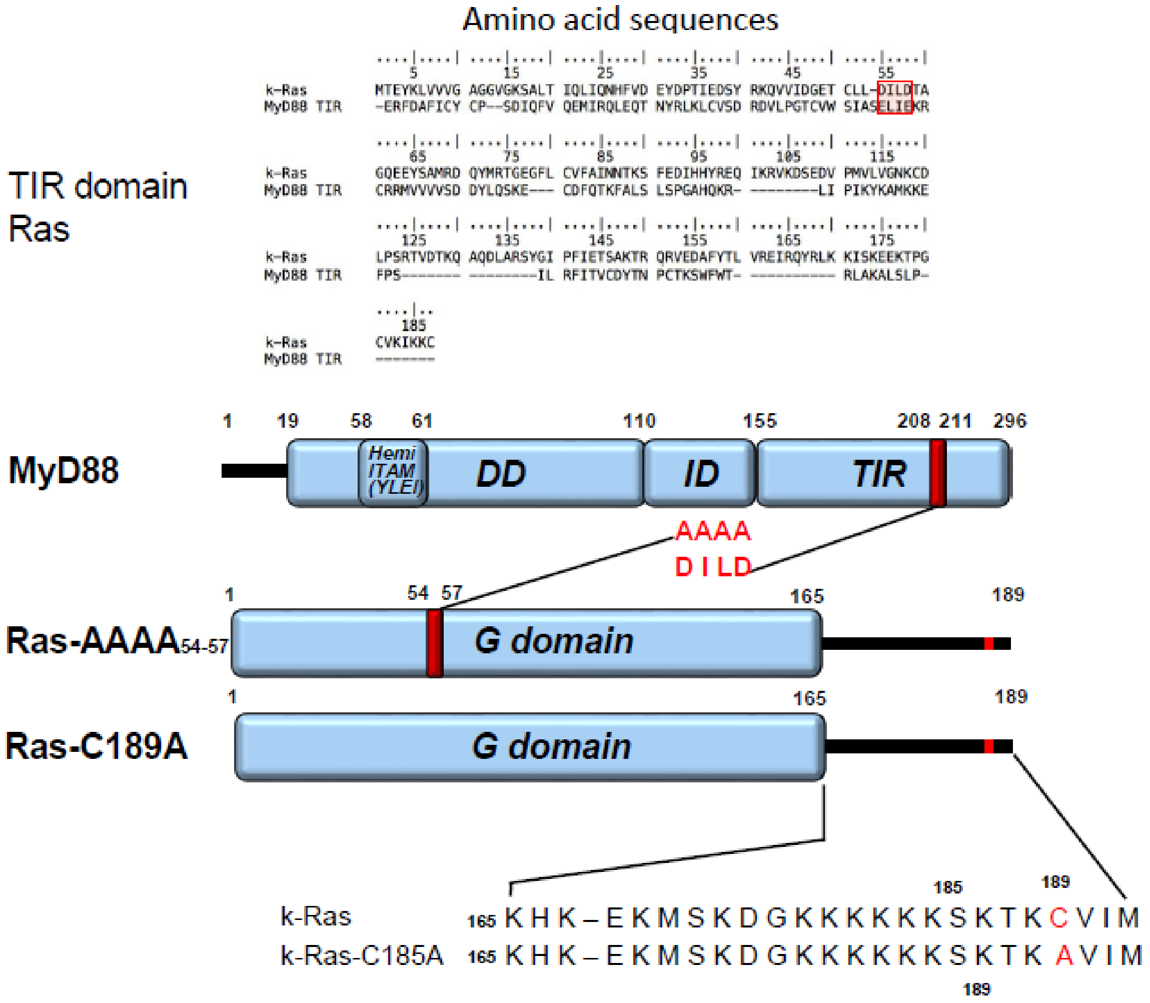

Figure S4. Amino acid sequence alignment between the TIR domain and Ras, and constructs of Ras and its mutants.

(A)

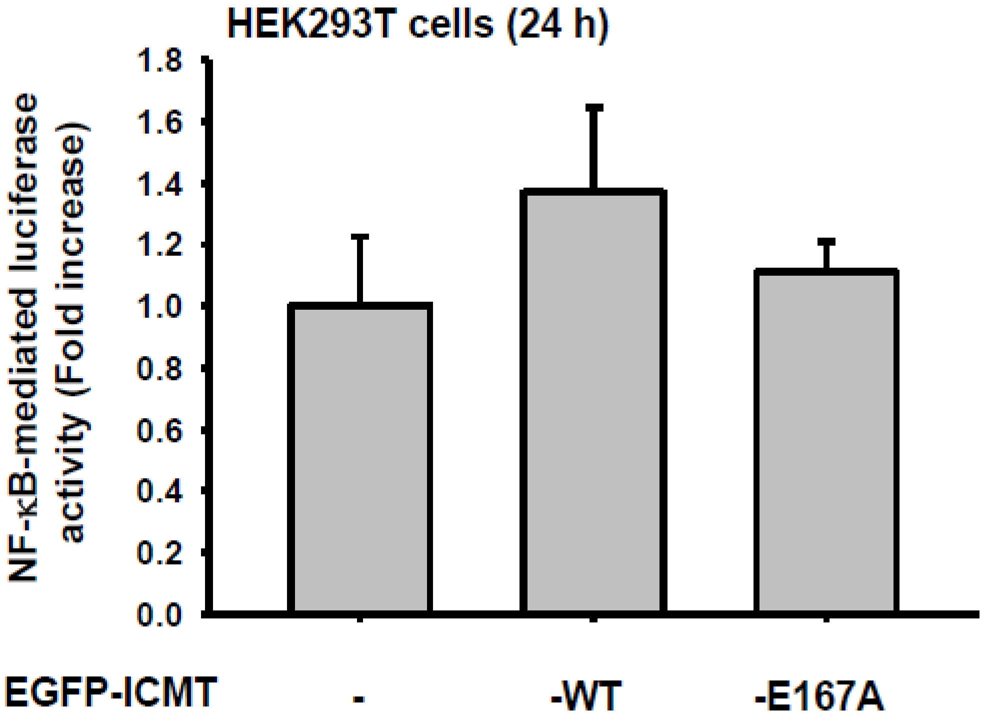
HEK293T cells (24 h)
NF-κB-mediated luciferase activity (Fold increase)
1.8
1.6
1.4
1.2
1.0
0.8
0.6
0.4
0.2
0.0
EGFP-ICMT
-
-WT
-E167A

(B)

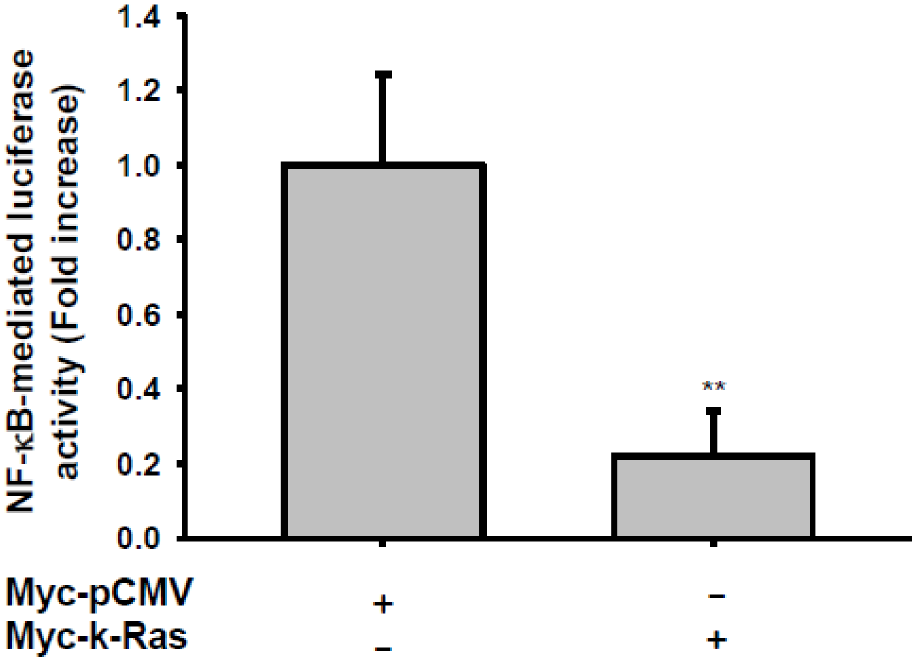

NF-κB-mediated luciferase activity (Fold increase)
1.4
1.2
1.0
0.8
0.6
0.4
0.2
0.0
**
Myc-pCMV
+
–
Myc-k-Ras
–
+

(C)

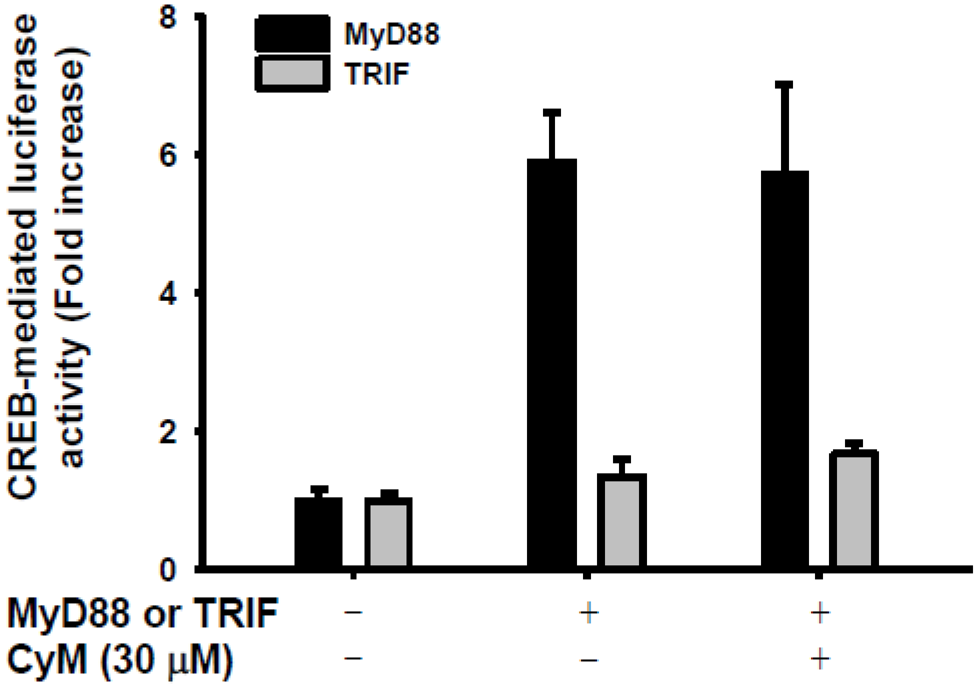

CREB-mediated luciferase activity (Fold increase)
8
6
4
2
0
MyD88
TRIF
MyD88 or TRIF − + +
CyM (30 μM) − − +

(D)

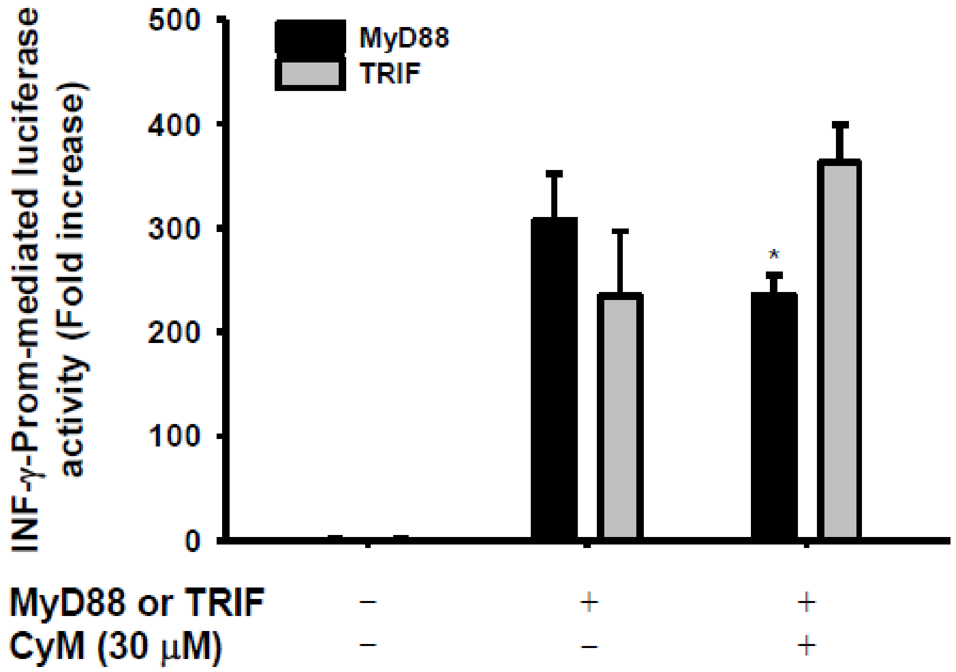
INF-γ-Prom-mediated luciferase activity (Fold increase)
500
400
300
200
100
0
MyD88
TRIF
*
MyD88 or TRIF − + +
CyM (30 μM) − − +

(E)

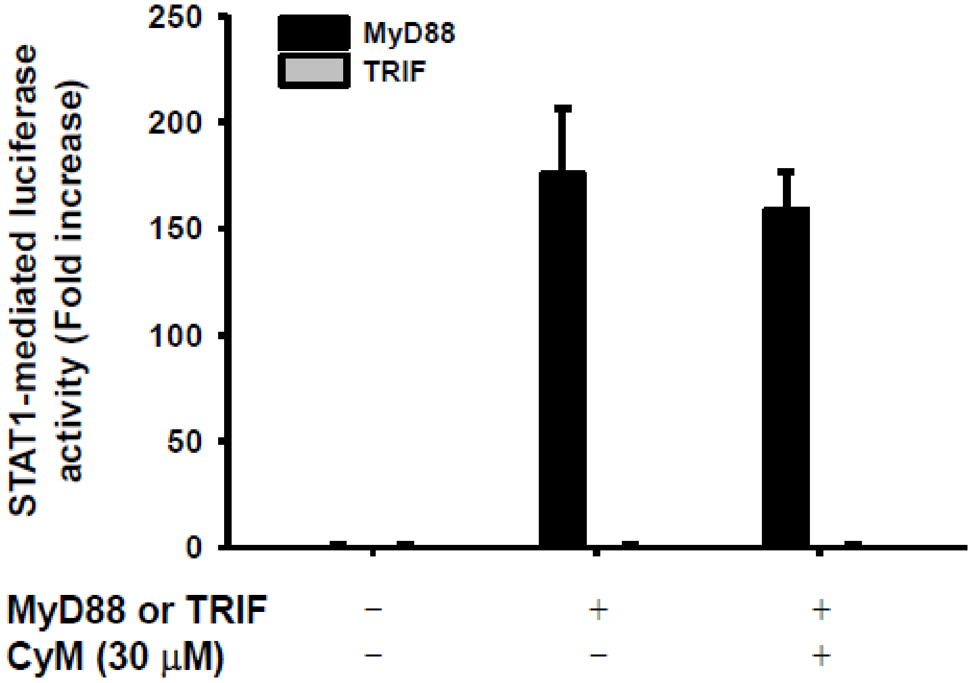
STAT1-mediated luciferase activity (Fold increase)
250
200
150
100
50
0
MyD88
TRIF
MyD88 or TRIF − + +
CyM (30 μM) − − +

(F)

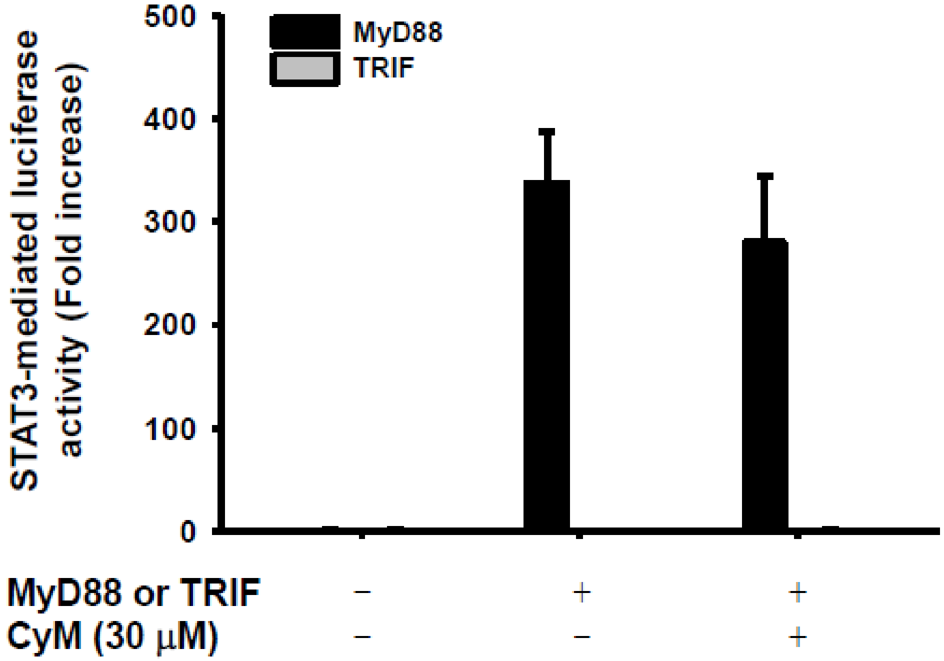

Figure S5. Effect of ICMT on the induction of activities of NF-κB, CREB, STAT1, STAT3, and other transcription factors (NFAT, AP-4, YY-1, ATF, T-bet, and GATA). (A, B, C, D, E, and F) Induction effect of ICMT or Ras on luciferase activity induced by NF-κB, CREB, STAT1, and STAT3 in HEK293 cells transfected with ICMT (A), Ras (B), or adapter molecules (MyD88 and TRIF) (C, D, E and F) in the presence or absence of CyM (30 μM) (C, D, E and F) were analyzed using reporter gene assay with luminometer. *: $p < 0.05$ and **: $p < 0.01$ compared to empty vector control group.

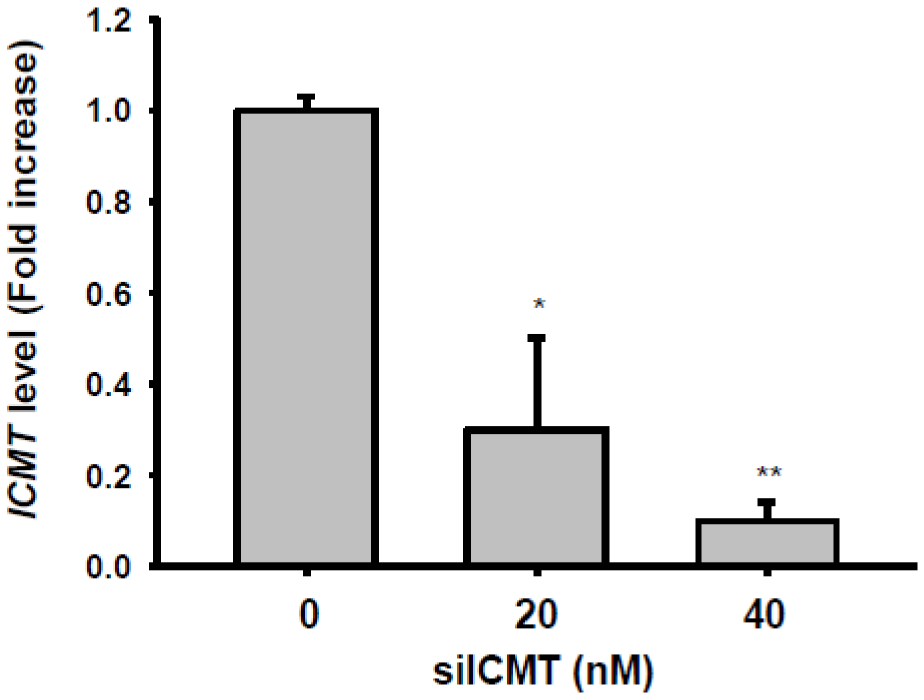

Figure S6. The mRNA expression level of *ICMT* in RAW264.7 cells transfected with siICMT. The mRNA level of *ICMT* from siICMT-transfected RAW264.7 cells was analyzed using real-time PCR analysis. *: $p < 0.05$ and **: $p < 0.01$ compared to control transfection group.

(A)

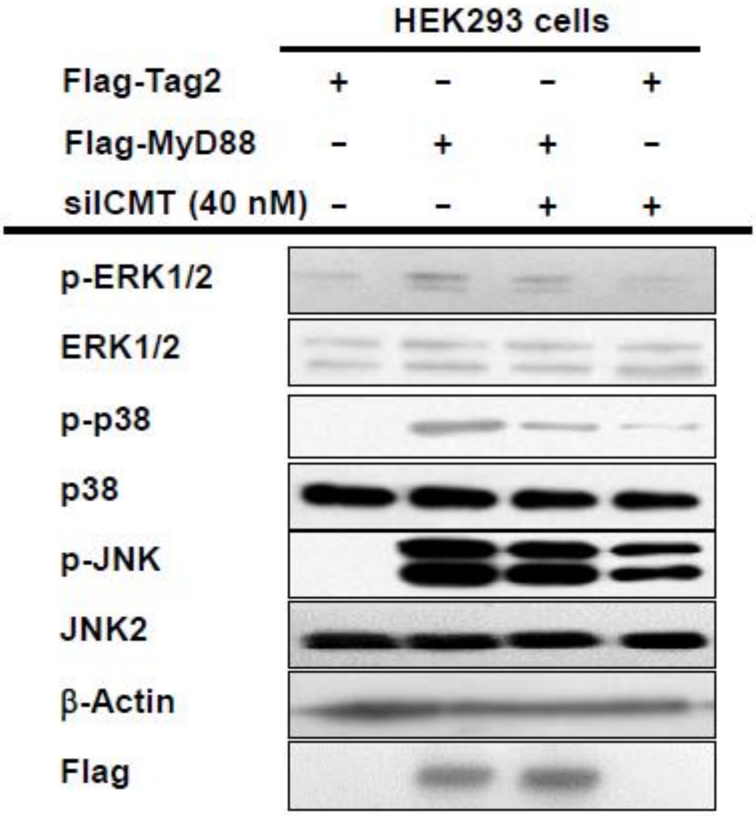
HEK293 cells
Flag-Tag2 + – – +
Flag-MyD88 – + + –
siICMT (40 nM) – – + +
p-ERK1/2
ERK1/2
p-p38
p38
p-JNK
JNK2
β-Actin
Flag

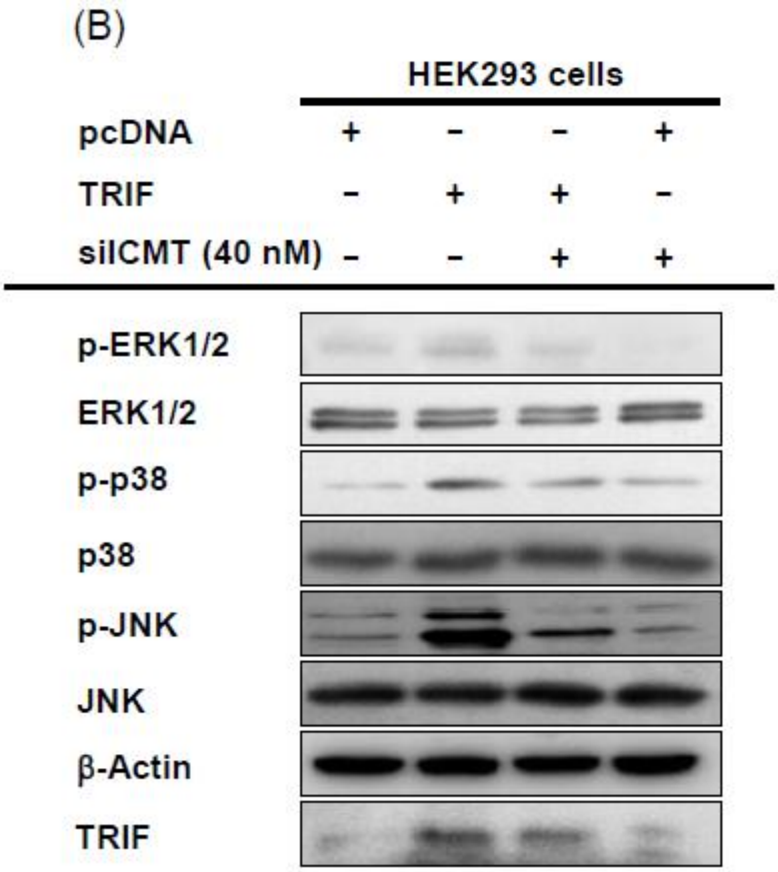

Figure S7. Effect of ICMT on the activation of MAPK in HEK293 cells transfected with MyD88 or TRIF. (A and B) Total and phospho-protein levels of MAPKs (ERK1/2, JNK1/2, and p38) in HEK293 cells transfected with MyD88 (A) and TRIF (B) in the presence or absence of siICMT were analyzed using immunoblotting analysis.

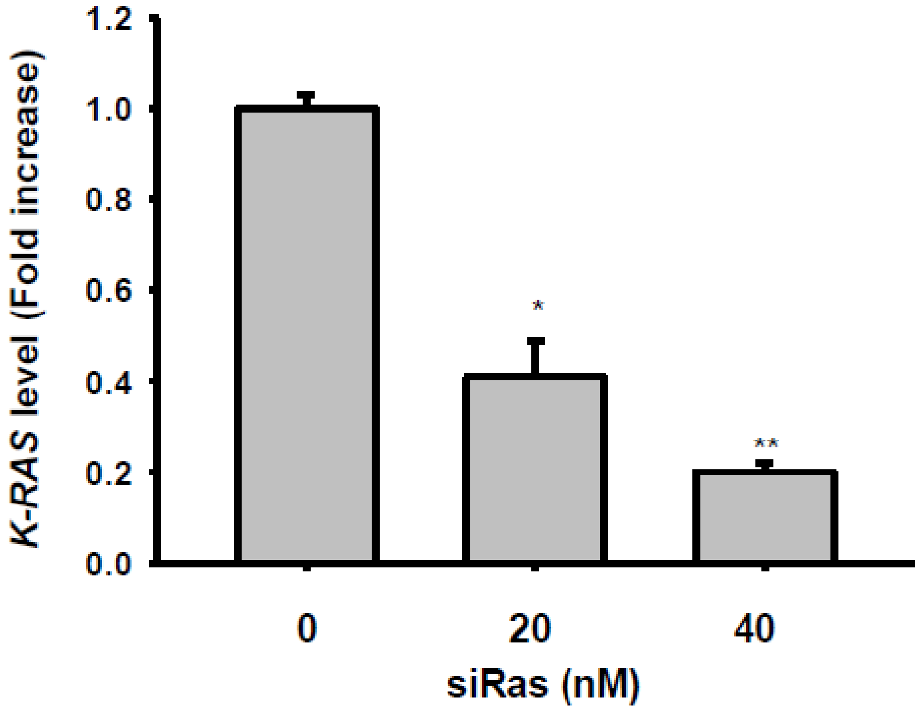

Figure S8. Effect of siRAS on the knockdown level of *K-RAS* in HEK293 cells. Gene expression levels of *K-RAS* in HEK293 cells transfected with siRas were analyzed using real-time PCR analysis. *: $p < 0.05$ and **: $p < 0.01$ compared to the control siRNA transfection group.

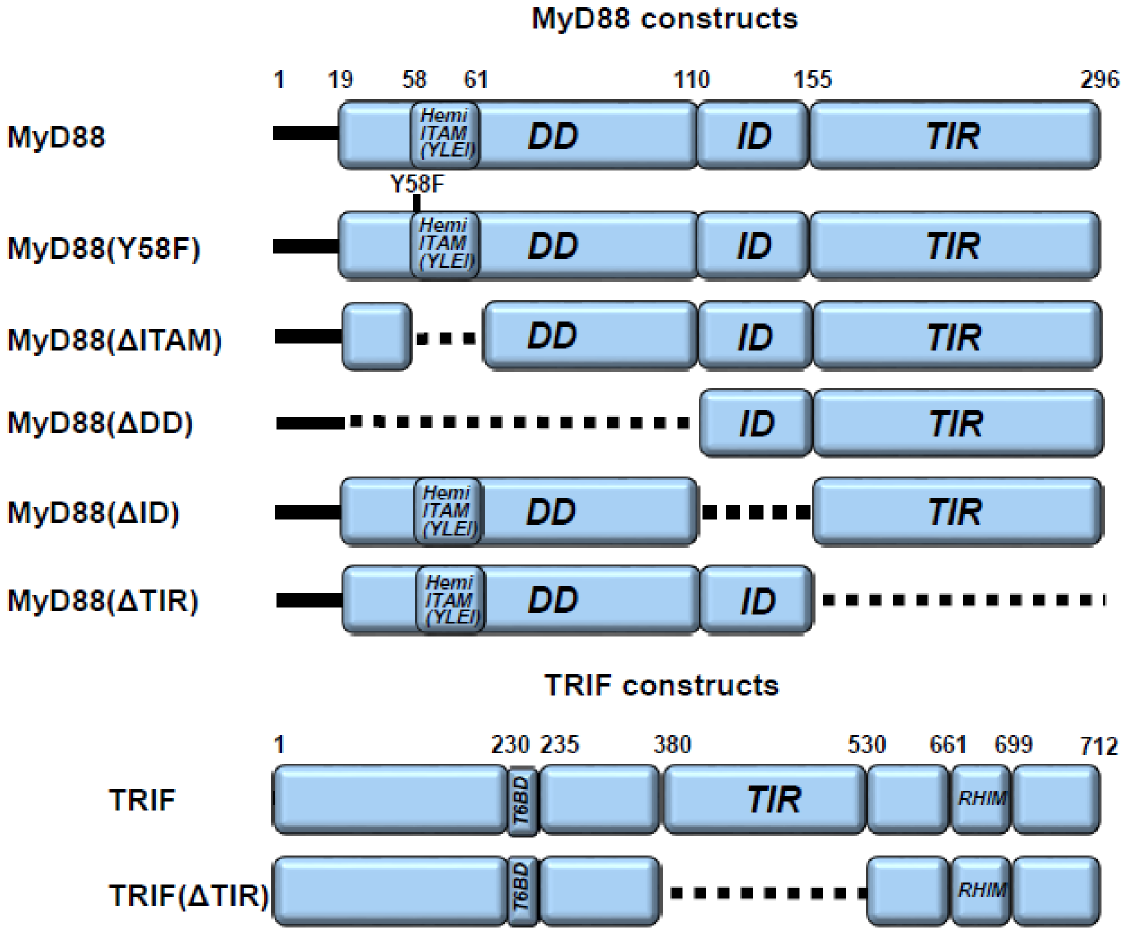

Figure S9. Constructs of *MyD88* and *TRIF* and their mutant forms.
